# Active transportation and public transportation use to achieve physical activity recommendations? A combined GPS, accelerometer, and mobility survey study

**DOI:** 10.1186/s12966-014-0124-x

**Published:** 2014-09-27

**Authors:** Basile Chaix, Yan Kestens, Scott Duncan, Claire Merrien, Benoît Thierry, Bruno Pannier, Ruben Brondeel, Antoine Lewin, Noëlla Karusisi, Camille Perchoux, Frédérique Thomas, Julie Méline

**Affiliations:** Inserm, UMR_S 1136, Pierre Louis Institute of Epidemiology and Public Health, Faculté de Médecine Saint-Antoine, 27 rue Chaligny, 75012 Paris, France; Sorbonne Universités, UPMC Univ Paris 06, UMR_S 1136, Pierre Louis Institute of Epidemiology and Public Health, Faculté de Médecine Saint-Antoine, 27 rue Chaligny, 75012 Paris, France; Département de Médecine Sociale et Préventive, Université de Montréal, 1430 Boulevard Mont-Royal, Montréal, Québec H2V 4P3 Canada; Human Potential Centre, Auckland University of Technology, 17 Antares Place, Mairangi Bay, Auckland, 0632 New Zealand; Centre d’Investigations Préventives et Cliniques, 6 rue La Pérouse, 75116 Paris, France; EHESP School of Public Health, Avenue du Professeur Léon Bernard, 35043 Rennes, France

**Keywords:** GPS, Accelerometry, Physical activity, Energy expenditure, Transportation, Walking, Public transportation

## Abstract

**Background:**

Accurate information is lacking on the extent of transportation as a source of physical activity, on the physical activity gains from public transportation use, and on the extent to which population shifts in the use of transportation modes could increase the percentage of people reaching official physical activity recommendations.

**Methods:**

In 2012–2013, 234 participants of the RECORD GPS Study (French Paris region, median age = 58) wore a portable GPS receiver and an accelerometer for 7 consecutive days and completed a 7-day GPS-based mobility survey (participation rate = 57.1%). Information on transportation modes and accelerometry data aggregated at the trip level [number of steps taken, energy expended, moderate to vigorous physical activity (MVPA), and sedentary time] were available for 7,644 trips. Associations between transportation modes and accelerometer-derived physical activity were estimated at the trip level with multilevel linear models.

**Results:**

Participants spent a median of 1 h 58 min per day in transportation (8.2% of total time). Thirty-eight per-cent of steps taken, 31% of energy expended, and 33% of MVPA over 7 days were attributable to transportation. Walking and biking trips but also public transportation trips with all four transit modes examined were associated with greater steps, MVPA, and energy expenditure when compared to trips by personal motorized vehicle. Two simulated scenarios, implying a shift of approximately 14% and 33% of all motorized trips to public transportation or walking, were associated with a predicted 6 point and 13 point increase in the percentage of participants achieving the current physical activity recommendation.

**Conclusions:**

Collecting data with GPS receivers, accelerometers, and a GPS-based electronic mobility survey of activities and transportation modes allowed us to investigate relationships between transportation modes and physical activity at the trip level. Our findings suggest that an increase in active transportation participation and public transportation use may have substantial impacts on the percentage of people achieving physical activity recommendations.

**Electronic supplementary material:**

The online version of this article (doi:10.1186/s12966-014-0124-x) contains supplementary material, which is available to authorized users.

## Background

An emerging field of research focuses on the relationships between transportation and physical activity and health [1]. The available evidence suggests that walking or cycling for transportation is beneficial for body weight and cardiovascular health [2,3]. However, there are questions relevant to public health policy makers, physicians, and health counselors that have yet to be answered. First, more accurate data are required to elucidate the contribution of transportation to daily physical activity. Even the exact gain in minutes of moderate to vigorous physical activity (MVPA) resulting from walking for transportation for a certain time (e.g., 10 minutes) in the typical conditions of a given transportation system is not known. Second, accurate information on the physical activity gains to be made from a wider use of public transportation is still lacking [4]. Third, the extent to which population shifts in the use of transportation modes could affect the percentage of people achieving physical activity recommendations is relatively unexplored [5]. These data would be useful to determine the extent to which promoting active transportation can raise physical activity levels in the population, but are difficult to derive without a relatively complex data collection strategy as the one proposed here.

Accurate evidence is lacking, as most studies have relied on retrospective survey questionnaires to assess mobility/transportation [6-10] or physical activity [7-9]. While a retrospective assessment of physical activity leads to measurement error of around 35-50% [11], accelerometry appears as an efficient way to monitor transportation physical activity. Similarly, Global Positioning System (GPS) technologies are receiving an increasing interest because retrospective mobility surveys often yield imprecise information on departure/arrival times of trips and lead to an underreporting of short walking trips [12], underestimation of car travel times, and overestimation of public transportation travel times [13].

Recent studies from separate fields have relied on GPS receivers to assess mobility. Public health researchers have combined GPS receivers and accelerometers to assess the environmental contexts of physical activity [14-16]. However, they lacked systematic and accurate information on the transportation modes used in each trip. To address this concern, the RECORD GPS Study [17,18] (as an alliance between Public health and Transportation sciences) is the first ever to combine over 7 consecutive days GPS tracking and accelerometers with an exhaustive mobility survey of transportation modes based on the presentation of GPS tracks [19,20]. The precision in the data makes it possible to analyze transportation physical activity not only at the individual level but also more accurately at the trip level. These data from a European city gave us the opportunity to investigate relationships between transportation modes (including public transportation) and physical activity, and to assess the impact of shifts in transportation modes on the achievement of physical activity recommendations.

## Methods

### Data collection and processing

#### Population

The RECORD participants, recruited during preventive health checkups in 2007–2008 and 2011–2013, were born in 1928–1978 and were residing (at baseline) in 112 municipalities of the Paris Ile-de-France region. In the second study wave [21-26], after undergoing a medical checkup and completing computerized questionnaires at the IPC Medical Centre [27], 410 participants were invited, through a standardized information and recruitment form, to enter the RECORD GPS Study (approved by the French Data Protection Authority) between February 2012 and June 2013. Of these, 247 accepted to participate, and signed an informed consent form. Nine participants withdrew from the study and data were incomplete for four participants, resulting in a final participation and completion rate of 57.1% (N = 234).

#### Collection of GPS and accelerometer data

Participants wore a QStarz BT-Q1000XT GPS receiver [28] and an Actigraph GT3X + tri-axial accelerometer [29] on the right hip with a dedicated elastic belt for the recruitment day and 7 additional days. They were asked to remove the belt only when sleeping and when they were in contact with water. Participants were instructed to recharge the GPS battery overnight, and to complete a travel diary of their activity locations (with arrival and departure times) over the data collection period, as supporting information for the electronic mobility survey. Two phone calls were made to the participants to reinforce the instructions.

#### Preprocessing of GPS data

The GPS data (one point every 5 seconds) were processed prior to the mobility survey by an ArcInfo 10 Python script [30]. The aim was to identify the participants’ activity locations (any type of activity at a stationary location) over the data collection period from the accumulation of GPS datapoints at certain locations. The algorithm calculates a kernel density surface based on the set of GPS points for each participant, extracts peaks as potentially visited locations, and derives a list of all visits over the period made to each detected location with their start and end times (Additional file 1: Appendix 1).

The algorithm automatically uploads the history of visits to locations into the server database of the electronic survey application and generates a set of pdf maps of the tracks that were mailed to the participants prior to the mobility survey.

#### GPS-based mobility survey

The telephone mobility survey was based on the Mobility Web Mapping application (Additional file 1: Appendix 2). With the help of the participants, the survey operator had to geolocate visits to activity locations undetected by the algorithm or for which GPS data were missing, and could modify/remove detected visits to locations that were inaccurate/incorrect. The dates/hours of arrival/departure to/from locations were provided by the algorithm but could be edited. The information collected for each visit to locations included the type of activity practiced at the location and the different transportation modes (19 options) that were used to arrive at the location in a chronological order.

In the present study, a trip corresponds to the travel from one destination to the next destination. Destinations correspond to places where people fulfill certain functions. Based on the terminology in the transportation sciences [31], trips are often performed with different transportation modes (e.g., walking and public transportation), and trip stages refer to the unimodal portions of trips (segments of trips based on a unique mode).

#### Post-processing of GPS and survey data

Survey mobility data were automatically checked by a custom SAS program. A report of errors (Additional file 1: Appendix 3) was edited for correcting survey data until no inconsistency remained in the data. The SAS program generated a detailed timetable over 7 days indicating the succession of activity locations and trips between locations with the start/end times of each episode and the corresponding information on activities and transportation modes. This timetable integrates information extracted by the algorithm from the GPS data, and the corrections, additions, and attribute data from the survey. A dummy variable was generated to indicate whether the start and/or end times of each trip were derived from the automatic processing of GPS data or collected/corrected during the survey.

#### Aggregation of accelerometer data

ActiLife 5.10 with default settings was used to identify episodes of nonwear of the accelerometer (floating windows of consecutive epochs with a 3-axes count equal to 0 for at least 60 min with a Spike tolerance of 2 min of nonzero epochs). Trips that overlapped a nonwear period were flagged. The following accelerometer variables were aggregated for each trip or each visit to locations over 7 days according to the start/end times of the episode: (i) number of steps taken; (ii) time spent in MVPA as the sum of the 5-second epochs with a 3-axes number of counts ≥2,690/12 [29]; (iii) time spent sedentary (counts per min <150, either determined on an epoch or min basis) [32]; and (iv) energy expenditure in kcal calculated from activity counts and participants’ weight from three formulas: the Sasaki and Freedson equation [29]; the refined Crouter equation [33]; and the “Freedson VM3 Combination” equation from the Actigraph website [34]. Regarding sedentary time, the first approach separately used the data of each 5-second epoch, multiplied each axis counts by 12 to rescale them at the minute level, calculated the rescaled vector magnitude on this basis, and flagged the 5-second epoch as sedentary if the rescaled vector magnitude counts were less than 150. Differently, the second approach aggregated 12 successive 5-second epochs into minute epochs and flagged a minute as sedentary if the cumulated counts were below 150. Details are provided in Additional file 1: Appendix 4.

Default processing of GT3X + data involves a filter limiting the sampling to the frequency range of 0.25-2.5 Hz to exclude nonhuman accelerations. An optional low-frequency extension filter extends the lower end of the filter (useful when processing data of people who move slowly) [35]. Analyses were conducted both with the normal filter and with the low frequency extension filter activated (Additional file 1: Appendix 5).

#### Study variables

Trip-level accelerometry outcomes were analyzed: (i) directly as obtained from the aggregation of data at the trip level; and (ii) standardized for trip length (expressed per 10 min or per km traveled) prior to the modeling.

As a proxy of the distance covered during trips, the present article relies on the length in m of the shortest path between the origin and the destination of the trip through the street network (determined with ArcInfo 10 and street data of the National Geographic Institute).

The transportation mode variable was defined only among trips made with a unique mode or with a unique mode in addition to walking. We had to exclude trips with two or more nonwalking modes because they could not be attributed to the mutually exclusive categories of modes that were needed to perform the comparison, and because there were much too few trips with each combination of two nonwalking modes to define additional categories. The simpler version of the variable distinguished: walking only, biking, public transportation, and personal motorized vehicle. A more detailed variable subdivided public transportation into: bus/coach; metro (available in Paris and immediate surroundings); RER (fast trains traveling through the suburbs), train, or TER (trains from Paris towards suburbs or adjacent regions); and tramway. Personal motorized vehicle was subdivided into driving a four-wheel motorized vehicle; being the passenger of a four-wheel motorized vehicle (including taxi); and using a two-wheel motor vehicle.

### Analysis

#### Relationships between transportation modes and physical activity

Additional file 1: Appendix 6 provides summary information on the sample size in which each statistic was calculated and each analysis performed (each statistic was calculated in the sample in which it was the most meaningful, e.g., summary information on trips in the largest sample). The regression analyses excluded certain trips from the full sample (n = 7,644) to improve the meaningfulness of the analyses. First, data on the start time and end time of each trip were validated at the minute level during the mobility survey. Accordingly, trips of less than 1 minute often had a 0 minute duration in the final database. By definition, such trips (n = 218) could not be used to aggregate accelerometry data and were thus excluded from the analyses. The following trips were also excluded: trips that overlapped a period of nonwear of the accelerometer (n = 480); trips of less than 5 m of length (n = 53); trips that started and ended at the same location (n = 10); recreational walking trips or atypical trips (professional tours, etc.) (n = 22); trips with an excessive duration compared with the distance covered and the mode used (Additional file 1: Appendix 7) (n = 62); trips starting and/or ending out of the Ile-de-France region (n = 509); trips with another transportation mode than in the classification employed (n = 63); and trips that included two or more nonwalking modes. The latter criterion led to the exclusion of a different number of trips (n = 63 or n = 360) depending on the classification of modes (crude or detailed) that was used, resulting in samples of 6,164 and 5,867 trips for the crude and detailed classifications. With the crude classification, 77% of the trips with multiple modes that were excluded used both a personal motorized vehicle and public transport while 11% of the trips relied on the use of both a bike and a personal motorized vehicle.

Multilevel linear models, applied to data at the trip level with a random effect at the individual level, were used to estimate associations between the transportation mode in each trip and the trip-level accelerometry variables (raw, time-standardized, and distance-standardized outcomes).

The study was interested in unadjusted trip-level relationships between transportation modes and physical activity, for which confounding is unlikely (see Additional file 1: Appendix 8). As secondary analyses, models were adjusted for trip-level and individual-level variables as main effects and interacting with transportation modes, as estimates of the transportation mode–physical activity relationship by subgroups of trips and individuals (reported in Additional file 1: Appendix 8).

Regression models were finally rerun after excluding trips for which the start and/or end times were edited or generated during the survey (as opposed to determined by the algorithm).

#### Scenarios of shifts in transportation mode use

Two hypothetical scenarios of shift of transportation mode use were examined (Table 1). Each eligible trip to a shift of transportation mode (see also bottom of Table 1) was assigned a probability of shift. This probability of shift was equal to the basal probability from the scenario, plus an individual term, plus a penalty term. The individual term was a random value between −0.1 and +0.1 on the probability scale to account for the fact that participants changing mode for one of their trips would have a greater likelihood to make a comparable change for their other trips. For shifts from personal motorized vehicle to public transportation, a penalty of 0.05, 0.1, 0.1, and 0.2 on the probability scale was applied, respectively, to trips between Paris and the suburb/provinces; trips from the suburb/provinces to the suburb/provinces; trips on a Sunday; and trips between 0:00 am and 5:00 am. Justifications for these parameters are reported in Additional file 1: Appendix 9. For trips with a personal motorized mode that were eligible to a change to both walking and public transportation, a multinomial probability was calculated, with the overall probability of change equal to the sum of the probabilities of change to walking and public transportation. Probabilities of change below 0 were set to 0, and those above 1 were set to 1.

**Table 1:** **Two hypothetical scenarios of shift in transportation modes** ^**a**^

**Trips targeted by the scenarios**	**Scenario 1**	**Scenario 2**
**Trips <1 km**		
By personal motorized vehicle	30% to walking	60% to walking
	15% to public transportation	30% to public transportation
By public transportation	30% to walking	60% to walking
**Trips between 1 and 2 km**		
By personal motorized vehicle	15% to walking	30% to walking
	20% to public transportation	40% to public transportation
By public transportation	15% to walking	30% to walking
**Trips between 2 and 3 km**		
By personal motorized vehicle	7.5% to walking	15% to walking
	25% to public transportation	50% to public transportation
By public transportation	7.5% to walking	15% to walking
**Trips between 3 and 5 km**		
By personal motorized vehicle	25% to public transportation	50% to public transportation
**Trips between 5 and 10 km**		
By personal motorized vehicle	20% to public transportation	40% to public transportation
**Trips between 10 and 50 km**		
By personal motorized vehicle	15% to public transportation	30% to public transportation

^a^The following trips, which were excluded from the modeling of relationships between transportation modes and physical activity, were defined as eligible for a shift of mode: (i) trips that were entirely included in or partly overlapped a period of nonwear of the accelerometer; and (ii) trips that started and/or ended out of the Ile-de-France region. Trips that were not eligible for a shift of mode included: trips of 0 min of length in the database; trips of less than 5 m of length; trips that started and ended at the same location; recreational walking trips or particularly atypical trips; trips with an excessive duration compared to the distance covered and to the mode used; trips with another transportation mode than those in the classification employed; and trips that included two or more nonwalking modes.

A random number between 0 and 1 was drawn for each eligible trip. A shift of mode occurred if the random number was below the probability of change. For each trip with a shift of mode, the original number of min of MVPA was replaced by the predicted number of min of MVPA with the new mode. The prediction was based on the final model including interactions between trip-level or individual-level variables and transportation modes.

The experiment was replicated 1000 times (by randomly drawing 1000 sets of numbers between 0 and 1). For each replicate, the percentage of participants with ≥210 min of MVPA over 7 days was determined (≥30 min per day, according to the official physical activity recommendation [36]), taking into account both periods at activity locations and trips. The increase in the percentage of participants reaching the recommendation from the observed data to the scenario examined was calculated for each replicate. The 1000 replicates permitted to derive a 95% credible interval (CrI) for the increase in percentage.

## Results

The median age of participants was 58 [interdecile range (IR): 41 – 73]. In the sample, 65% of the participants were males; 52% were employed, 2% unemployed, and 40% retired; and 97% had a driving license, 88% had access to a 2-wheel or 4-wheel personal motorized vehicle in their household, and 39% had a public transportation pass. Participants had a median body mass index of 24.9 kg/m^2^ (IR: 21.0 – 30.5) based on measured height and weight.

### Accelerometer wear time and time with valid GPS data

The daily accelerometer wear time per participant over the 7 days had a median value of 12 h 35 min (IR: 9 h 00 min – 14 h 47 min). The median number of days with ≥10 hrs of wear time per participant was 6 (respectively 4 and 7 for the first and ninth deciles). Seven percent of the trips and 31% of the episodes at activity locations overlapped a period of nonwear of the accelerometer. Using as a denominator the time over which the accelerometer (thus the belt with the GPS) was worn, the percentage of time with valid GPS data was of 65% (median value in the sample of 234 individuals), while the percentage of time with imprecise/invalid GPS data (HDOP ≥6, VDOP ≥7, or PDOP ≥8) was of 1% and the percentage of time without GPS data was of 33% (a shorter time of valid GPS data is not an indication of data of poorer quality as it might reflect a longer time spent indoor or in underground transportation modes; indeed, when there are less than 3 satellites available, the BT-Q1000XT stops logging data).

### Percentage of physical activity attributable to transportation

The median number of visits to locations (including the residence) per participant over the 7-day measurement period was 33 (IR: 20, 49). Participants spent a median of 8.2% (IR: 4.2%, 13.4%) of their time in transportation, corresponding to 1 h 58 min per day (IR: 1 hr 01 min, 3 hr 13 min).

Transportation, as opposed to episodes at activity locations, accounted for a median of 38% of the steps taken over 7 days (IR for the 234 participants: 16%, 58%). Similarly, transportation accounted for 31% of energy expenditure (IR: 12%, 50%) (Sasaki-Freedson equation, Additional file 1: Appendix 10 for other definitions), for 33% of MVPA time (IR: 12%, 52%), and for 13% of sedentary time (IR: 5%, 23%) (epoch-level definition). Additional file 1: Appendix 10 indicates the contribution of physical activity at activity locations and during trips to the variations between individuals in these percentages.

### Description of trips by transportation modes and lengths

**Figure 1:**
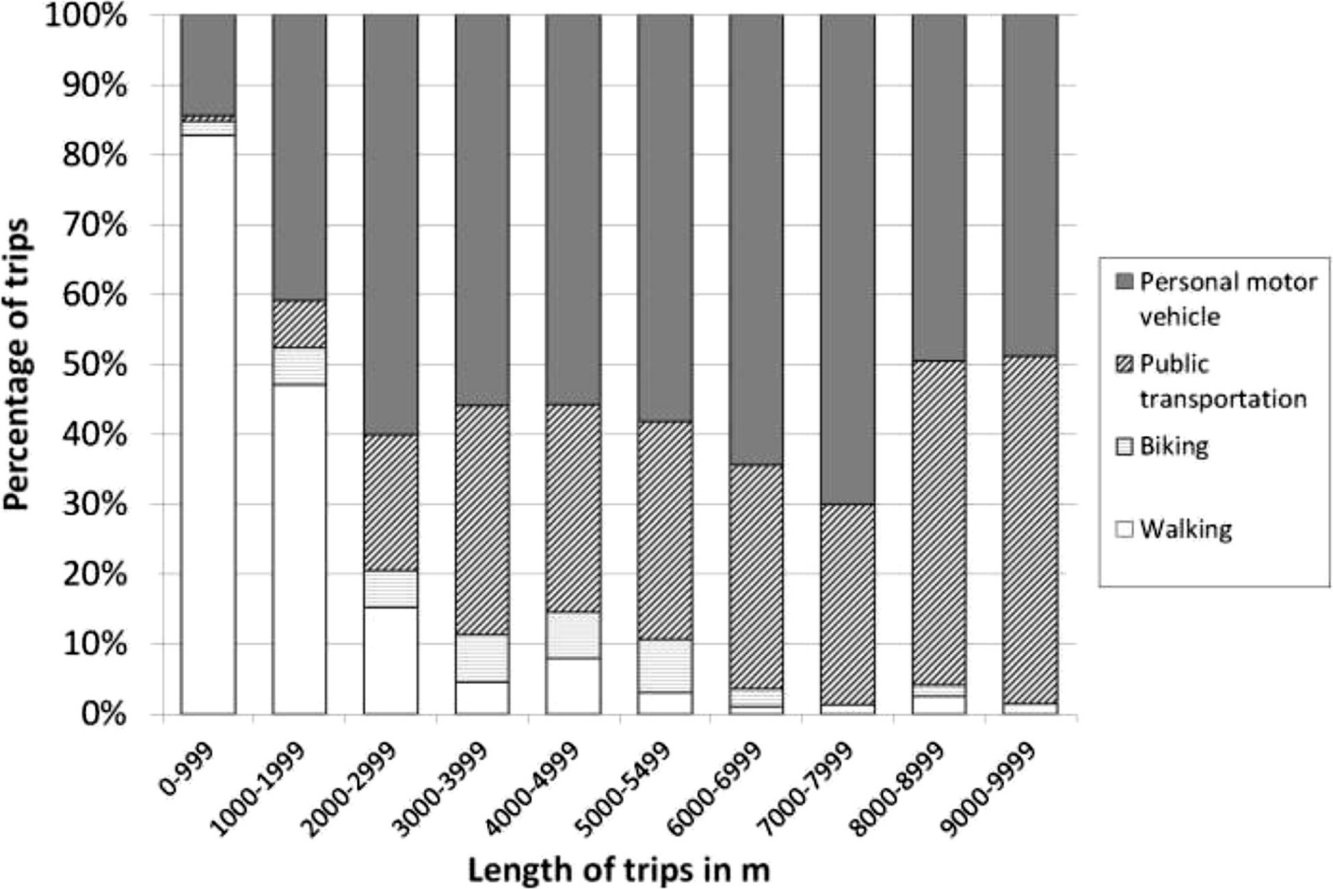
**Distribution of the transportation modes used according to the length of the trips in m.** The distribution was determined among single mode trips, after excluding trips made with an alternative mode.

The median street-network length of trips (n = 7,644) was 1,463 m (IR: 224, 15,029). Their median time length was 15 min (IR: 2 min, 1 hr 03 min). Among single mode trips, 44.0% were made by walking, 3.1% by biking, 38.4% by personal motorized vehicle, and 14.5% by public transportation. A steep decrease in the percentage of trips made by walking with increasing distance was observed, from over 80% for distances <1,000 m to around 5% for trips between 3,000 and 5,000 m in length (Figure 1). The percentage of trips by public transportation rather than by personal motorized vehicle was higher for trips >3000 m of length.

### Associations between transportation modes and physical activity

Unadjusted models indicated that walking, biking, and public transportation trips were associated with more steps taken and with more energy expended than trips by personal motorized vehicle (Table 2, unstandardized outcomes). Walking and public transportation trips were associated with greater MVPA. While biking and walking trips were associated with a lower sedentary time, public transportation trips were associated with a longer sedentary time than trips by personal motorized vehicle.

**Table 2:** **Trip-level associations between the transportation mode used and physical activity and energy expenditure (unstandardized outcomes) (n = 6,164 or 5,867 trips, N = 234 participants)** ^**a**^

**Transportation mode variable**	**Number of steps taken**	**MVPA (min)**	**Sedentary time (min)** ^**b**^	**Energy expenditure (kcal)** ^**c**^
	**β (95% CI)**	**β (95% CI)**	**β (95% CI)**	**β (95% CI)**
**Crude classification**				
Personal motorized vehicle	Ref.	Ref.	Ref.	Ref.
Public transportation	1210.9 (1140.6, 1281.2)	10.2 (9.6, 10.9)	11.7 (10.5, 12.8)	60.8 (57.0, 64.5)
Biking	180.7 (41.6, 319.8)	0.7 (−0.5, 1.9)	−9.5 (−11.8, −7.3)	10.8 (3.4, 18.3)
Walking	444.7 (393.0, 496.5)	3.8 (3.4, 4.3)	−11.2 (−12.0, −10.4)	22.6 (19.9, 25.4)
**Detailed classification**				
4-wheel motor, driving	Ref.	Ref.	Ref.	Ref.
4-wheel motor, passenger	9.9 (−115.3, 135.1)	– 0.0 (−1.1, 1.0)	2.7 (0.8, 4.6)	0.0 (−6.6, 6.7)
2-wheel motor vehicle	184.12 (−13.0, 381.3)	1.2 (−0.5, 2.0)	– 4.8 (−7.8, −1.9)	7.8 (−2.6, 18.3)
Metro	1038.3 (938.6, 1138.0)	9.2 (8.4, 10.1)	5.8 (4.2, 7.3)	52.3 (47.0, 57.6)
Bus/coach	773.8 (639.3, 908.2)	6.2 (5.1, 7.3)	4.4 (2.4, 6.5)	37.6 (30.5, 44.7)
Train	1596.3 (1442.3, 1750.4)	13.0 (11.7, 14.3)	12.7 (10.3, 15.0)	79.5 (71.4, 87.7)
Tramway	769.6 (426.3, 1112.9)	7.1 (4.1, 10.0)	– 0.5 (−5.8, 4.7)	35.6 (17.5, 53.8)
Biking	210.9 (72.1, 349.8)	0.9 (−0.3, 2.1)	– 9.2 (−11.3, −7.1)	11.9 (4.5, 19.2)
Walking	439.6 (385.2, 493.9)	3.8 (3.3, 4.2)	−11.5 (−12.4, −10.8)	22.3 (19.4, 25.2)

*CI* confidence interval, *MVPA* moderate to vigorous physical activity.

^a^The multilevel linear models included a random effect at the individual level. The crude and the detailed transportation mode variables were introduced in separate models.

^b^Each 5 second epoch was classified as sedentary or not (the regression coefficients were *a posteriori* converted in min of sedentary time). See Additional file 1: Appendix 11 for the findings on sedentary time directly determined on a min basis.

^c^Energy expenditure was calculated with the formula of Sasaki and Freedson. See Additional file 1: Appendix 11 for findings with other definitions.

Whether standardized by distance or time, walking trips were associated with a larger number of steps taken, greater MVPA, and more energy expended than trips by personal motorized vehicle (Table 3). Trips with all four public transportation modes were associated with more steps taken, greater MVPA, and more energy expended than trips by personal motorized vehicle when time-standardization was applied (1.6 to 2.9 more min of MVPA for each 10 min of trip depending on the public transportation mode). With distance-standardized outcomes, public transportation trips were associated with larger energy expenditure, but not with steps taken and MVPA.

**Table 3:** **Trip-level associations between the transportation mode used and physical activity and energy expenditure (time-standardized and distance-standardized outcomes) (n = 6,164 or 5,867 trips, N = 234 participants)** ^**a**^

**Transportation mode variable**	**Number of steps taken per 10 min or km of trip**	**MVPA per 10 min or km of trip (min)**	**Sedentary time per 10 min or km of trip (min)** ^**b**^	**Energy expenditure per 10 min or km of trip (kcal)** ^**c**^
	**β (95% CI)**	**β (95% CI)**	**β (95% CI)**	**β (95% CI)**
**Time-standardized outcomes**				
**Crude classification**				
Personal motorized vehicle	**Ref.**	**Ref.**	**Ref.**	**Ref.**
Public transportation	202.5 (177.0, 228.0)	1.8 (1.6, 2.0)	−0.6 (−0.8, −0.4)	10.7 (9.4, 12.1)
Biking	95.6 (45.1, 146.2)	0.4 (−0.1, 0.8)	−2.9 (−3.3, −2.5)	5.5 (2.8, 8.2)
Walking	500.7 (481.9, 519.5)	4.3 (4.1, 4.5)	−3.4 (−3.6, −3.3)	25.6 (24.6, 26.6)
**Detailed classification**				
4-wheel motor, driving	Ref.	Ref.	Ref.	Ref.
4-wheel motor, passenger	−2.7 (−49.9, 44.5)	0.0 (−0.4, 0.4)	0.2 (−0.2, 0.6)	0.9 (−1.6, 3.5)
2-wheel motor vehicle	12.9 (−61.0, 86.8)	−0.2 (−0.9, 0.4)	−1.6 (−2.2, −1.0)	−1.9 (−5.9, 2.1)
Metro	213.2 (175.7, 250.8)	1.9 (1.6, 2.3)	−0.9 (−1.2, −0.7)	11.5 (9.5, 14.5)
Bus/coach	193.5 (142.7, 244.3)	1.6 (1.1, 2.0)	−1.0 (−1.4, −0.6)	10.3 (7.6, 13.0)
Train	228.8 (170.7, 286.9)	1.9 (1.4, 2.4)	−0.7 (−1.2, −0.3)	12.3 (9.2, 15.4)
Tramway	292.9 (162.9, 422.8)	2.9 (1.7, 4.0)	−1.6 (−2.6, −0.6)	15.3 (8.4, 22.2)
Biking	94.5 (42.2, 146.7)	0.4 (−0.1, 0.8)	−2.9 (−3.3, −2.5)	5.5 (2.7, 8.3)
Walking	501.9 (481.5, 522.3)	4.3 (4.1, 4.5)	−3.5 (−3.7, −3.3)	25.7 (24.6, 26.8)
**Distance-standardized outcomes**				
**Crude classification**				
Personal motorized vehicle	Ref.	Ref.	Ref.	Ref.
Public transportation	122.4 (−13.9, 258.7)	1.2 (−0.1, 2.4)	0.7 (−1.1, 2.5)	7.9 (0.2, 15.5)
Biking	70.3 (−202.2, 342.8)	0.5 (−2.0, 3.0)	−0.8 (−4.4, 2.8)	4.3 (−10.9, 19.5)
Walking	1105.0 (1003.9, 1206.0)	9.6 (8.7, 10.6)	6.0 (4.6, 7.3)	58.1 (52.5, 63.8)
**Detailed classification**				
4-wheel motor, driving	Ref.	Ref.	Ref.	Ref.
4-wheel motor, passenger	60.7 (−197.8, 319.2)	0.6 (−1.7, 3.0)	1.3 (−2.1, 4.8)	4.3 (−10.1, 18.6)
2-wheel motor vehicle	−377.1 (−771.3, 17.2)	−3.8 (−7.4, −0.2)	−2.9 (−8.1, 2.3)	−37.5 (−59.6, −15.3)
Metro	137.2 (−68.3, 342.6)	1.4 (−0.5, 3.2)	1.1 (−1.7, 3.8)	8.3 (−3.2, 19.7)
Bus/coach	311.5 (30.5, 592.5)	2.7 (0.1, 5.3)	3.5 (−0.3, 7.3)	16.6 (1.1, 32.1)
Train	21.4 (−298.3, 341.1)	0.2 (−2.8, 3.1)	– 1.7 (−6.0, 2.6)	2.6 (−15.2, 20.3)
Tramway	30.2 (−697.7, 758.1)	0.8 (−5.9, 7.5)	0.2 (−9.6, 10.1)	4.0 (−36.0, 44.0)
Biking	59.7 (−223.6, 342.9)	0.4 (−2.2, 3.0)	– 0.8 (−4.5, 3.0)	3.3 (−12.5, 19.1)
Walking	1093.1 (983.2, 1202.9)	9.5 (8.5, 10.5)	6.0 (4.5, 7.4)	56.9 (50.8, 63.1)

*CI* confidence interval, *MVPA* moderate to vigorous physical activity.

^a^The multilevel linear models included a random effect at the individual level. The crude and the detailed transportation mode variables were introduced in separate models.

^b^Each 5 second epoch was classified as sedentary or not (the regression coefficients were *a posteriori* converted in min of sedentary time). See Additional file 1: Appendix 11 for the findings on sedentary time directly determined on a min basis.

^c^Energy expenditure was calculated with the formula of Sasaki and Freedson. See Additional file 1: Appendix 11 for findings with other definitions.

Analyses were rerun after removing trips whose start/end times had been corrected/provided during the survey (as opposed to detected by the algorithm and confirmed by the survey) (Additional file 1: Appendix 11). In this subsample of trips with presumably more reliable start/end times, larger differences in physical activity were observed between walking, biking, or public transportation use and relying on a personal motorized vehicle than in the total sample. In this subsample, public transportation use was associated with more steps taken, a longer MVPA, and larger energy expenditure than using a personal motorized vehicle, whether time- or distance-standardized outcomes were used.

### Scenarios of shift in transportation modes

Overall, 35.5% of the participants cumulated the recommended 210 min of MVPA over 7 days. With Scenario 1 (implying a shift in around 13.7% of all motorized trips), the percentage of participants reaching the recommendation increased by 5.6 points (95% CrI: 4.3, 7.3) to 41.0% (95% CrI: 39.7%, 42.7%). Scenario 2 (implying a shift in around 32.9% of all motorized trips) was associated with a 12.8 point increase (95% CrI: 10.7, 15.0) in the percentage of participants reaching the recommendation to 48.3% (95% CrI: 46.2%, 50.4%) of the participants. These two scenarios were associated with an average increase in MVPA over 7 days of respectively 13 min (95% CrI: 12 min, 14 min) and 31 min (95% CrI: 29 min, 32 min).

## Discussion

Our GPS receiver, accelerometer, and mobility survey study was able to accurately quantify the physical activity gains associated with walking for transportation or public transportation use compared to car driving. On this basis, the study could assess through simulations the extent to which scenarios of shift of transportation modes would increase the percentage of people reaching the official physical activity recommendation.

### Study limitations

First, apart from the fact that the participants were recruited without randomization, a sample of 234 participants could not adequately “represent” the complex transportation behavior of a background population of more than 5 million of residents. Although our large sample of trips included a diversity of trips with different transportation modes and various departure and arrival points (Paris and close and far suburbs), the recruitment procedure did not yield a sample that was representative of the trips of the background population. Factors influencing study participation could affect the relationships between transportation modes and physical activity. For example, if public transportation users from municipalities that are far rather than close from the recruitment center had lower odds to participate in the study, shorter public transportation trips would be overrepresented in the sample. As walking trip stages may represent a larger proportion of the trip in shorter than in long public transportation trips, such hypothetical selection process would distort the final estimate of the difference in physical activity between car use and public transportation use for each 10 min of trip.

A second limitation of the study is the intrinsic imprecision of a 7-day mobility survey, despite the presentation of GPS tracks to the participants to facilitate recall. Third, the exact distance covered during trips was not assessed; the shortest street network distance was used instead. Even if atypical trips were excluded, residual atypical trips for which the shortest distance is inaccurate may affect the predictive ability of the model for MVPA and the simulations.

Fourth, the estimated percentage of physical activity during non-sleeping time attributable to transportation was based on the assumption that nonwear periods only included sleeping or resting time. It was difficult to assess the direction of bias for such percentages, resulting from the relative importance of nonwear of the accelerometer during trips as opposed to during episodes with physical activity at given locations. Fifth, the simulations of scenarios of shift of transportation modes were based on a small sample at the individual level. Such simulations will have to be replicated with a large sample as available in transportation surveys (without precise 7-day GPS, accelerometry, and survey data as in the present study however).

Finally, accelerometers worn at the waist were unable to correctly quantify cycling-related physical activity [37]. This poor estimate did not permit to explore the impact of a shift of mode to biking in the scenarios examined for this population where the prevalence of cycling is low. Similarly, while the walk to one’s personal car or the longer walk to the public transportation stations should have been adequately captured, it is not clear whether the energy expenditure of other ambulatory activities such as climbing stairs in the subway was correctly evaluated by waist-worn accelerometers [38]. Moreover, apart from ambulatory activities, the physical activity and energy expenditure associated with a number of non-ambulatory activities, e.g., standing in a metro or in a bus for 20 minutes, may be poorly assessed through classical algorithms applied to waist-worn accelerometry. A final issue potentially distorting the estimated differences between transportation modes is that it is difficult to know whether nonhuman accelerations associated with motorized modes are entirely filtered out during the processing or whether part of these is in fact spuriously taken into account when calculating energy expenditure.

### Strengths of the study compared to previous literature

A previous study assessed physical activity gains from a shift from car to transit, but did not rely on accelerometry [5]. As recently reviewed [4], few studies have assessed the relationship between public transportation use and accelerometry-derived physical activity. However, the association was modeled at the individual rather than at the trip level, and was thus imprecise and vulnerable to confounding. One study conducted analyses at the trip level but did not use accelerometers [39]. Two studies using GPS receivers and accelerometers collected information on transportation modes, but did so only for a subset of the trips (e.g., only for home–school or home–work journeys) [40,41] or could not establish an exact time correspondence between the trips and accelerometry [40]. Overall, our study is the first to assess the physical activity gains to be made from walking or using public transportation with analyses conducted at the trip level based on data with the start and end times of trips from GPS data, with systematic information on transportation modes over 7 days from an electronic mobility survey, and with information on physical activity from accelerometers.

### Interpretation of findings

A substantial percentage of daily physical activity was made during transportation. It should be noted that this percentage was comparable (around 30%) for the number of steps taken, MVPA, and energy expenditure. The high share of physical activity attributable to transportation together with the between-individual variations in this percentage suggest that there may be substantial room to increase population physical activity levels through the promotion of active transportation.

It must be emphasized that it is more relevant to compare physical activity levels between transportation modes at the trip level (by considering entire trips possibly involving walking in addition to another transportation mode) than at the trip stage level (by considering the subparts of trips with a unique mode). Indeed, it is not particularly informative to compare physical activity levels when sitting in a car and when sitting in a bus or in a train, which levels are expected to be comparable; the interesting aspect in the comparison is that people often do not have to walk to use a car, while using public transportation implies to walk to the station and from the station to the arrival point of the trip.

The finding that walking for transportation (compared to using a personal motorized vehicle) was associated with 4.3 to 5.3 additional min of MVPA for each 10 min of trip is novel. Engaging in MVPA through walking implies walking at a certain speed; the extent to which walking for transportation in Ile-de-France leads to the accumulation of MVPA was not known. Additionally, public transportation use was associated with more physical activity than personal motorized vehicle use when unstandardized, time-standardized, and even distance-standardized (Additional file 1: Appendix 11) outcomes were used. While time standardization provides a physical activity perspective (practice of an activity for a given time), the distance standardization is compatible with a transportation perspective (traveling some distance).

The findings were provided at the trip level for each 10 min or km of trip to facilitate their use in future simulations assessing the physical activity and health impact of transportation or urban planning interventions intended to influence transportation habits. The present examination of scenarios of shift of transportation modes did not assess the feasibility of these scenarios and whether they were realistic or not. Our simulations suggest that increasing the percentage of trips made by walking or public transportation at the expense of car driving may be associated with a notable increase in the percentage of people reaching physical activity recommendations. However, it is important to emphasize that there may not be room for an increase in physical activity levels through transportation for everyone. Among participants who only engage in a low amount of physical activity through transportation, some may have the opportunity to walk or bike more often or to use public transportation more regularly. On the opposite, other people living in underserved areas (overrepresented among low socioeconomic status groups) may have to cover too long distances to reach services to be able to walk or bike there, and may live or work too far from public transportation stations to rely on this transportation mode. Simulation studies of scenarios of shift of modes will have to incorporate such social and environmental constraints into the modeling, in order to verify that promoting physical activity through transportation does not increase social disparities in physical activity levels, as may be anticipated.

Moreover, it must be emphasized that the overall percentage of physical activity made through the transport activity, the amount of physical activity accumulated when walking for transportation or using public transportation, and the gains in physical activity levels to be made from changes in transportation habits may vary from one city, region, or country to the other, due to differences in the configuration of the transportation system or in the transportation habits of populations.

## Conclusion

Overall, the present study is the first to assess the physical activity gains from walking or public transportation use with analyses at the trip level; with information on the start and end times of the trips from GPS data; with systematic information on transportation modes over 7 days from a mobility survey; and with physical activity data from accelerometers. With this innovative approach, we were able to accurately quantify differences in physical activity, steps taken, energy expenditure, and sedentary time between walking, using public transportation, and car driving, for each 10 min or km of trip.

Although future research should pay attention to populations facing social and environmental barriers, our assessment of scenarios of shift of transportation modes suggests that promoting walking and public transportation may have a substantial population-level impact on the percentage of people reaching physical activity recommendations. These European data provide further evidence on the benefits of active transportation, including public transportation use, as a strategy to promote physical activity and fight the obesity epidemic.

## Additional file

Additional file 1:
**Online Appendices.**

## Abbreviations

CI: Confidence interval
GPS: Global positioning system
IR: Interdecile range
MVPA: Moderate to vigorous physical activity
