# Supplementary material for: Active transportation and public transportation use to achieve physical activity recommendations? A combined GPS, accelerometer, and mobility survey study
Source: Int J Behav Nutr Phys Act. 2014 Sep 27;11:124. doi: 10.1186/s12966-014-0124-x (PMC4181295; doi:10.1186/s12966-014-0124-x)
Supplement: Additional file 1: — Online Appendices. [file 12966_2014_124_MOESM1_ESM.doc]

**Additional file 1: Online Appendices**

**Active transportation and public transportation use to achieve physical activity recommendations? A combined GPS, accelerometer, and mobility survey study**

[Appendix 1: Algorithm of preprocessing of GPS data 2](#__RefHeading___Toc390455042)

[Appendix 2: Application for the GPS-based prompted recall mobility survey 3](#__RefHeading___Toc390455043)

[Appendix 3: Verification of the mobility survey data 5](#__RefHeading___Toc390455044)

[Appendix 4: Management of accelerometry data 7](#__RefHeading___Toc390455045)

[Appendix 5: Analyses with the accelerometry low frequency extension filter activated 9](#__RefHeading___Toc390455046)

[Appendix 6: Definition of the samples of trips in which each analysis was performed and related sample sizes 11](#__RefHeading___Toc390455047)

[Appendix 7: Exclusion of trips of an excessive length 12](#__RefHeading___Toc390455048)

[Appendix 8: Models of relationships between transportation modes and physical activity adjusted for trip-level and individual-level variables 13](#__RefHeading___Toc390455049)

[Appendix 9: Justifications for the values of parameters to determine the probability of change of mode in each trip in the simulations 18](#__RefHeading___Toc390455050)

[Appendix 10: Percentage of physical activity attributable to transportation 19](#__RefHeading___Toc390455051)

[Appendix 11: Additional models estimated for the accelerometry outcomes 20](#__RefHeading___Toc390455052)

# Appendix 1: Algorithm of preprocessing of GPS data

The algorithm is developed in Python language and is released as an ArcToolBox for ArcGIS 10 . It relies on the spatial coordinates, timestamp, and dilution of precision indicators of the GPS data file. A preliminary task of the algorithm is to clean the GPS data by discarding observations with a poor signal quality (HDOP ≥6 or VDOP ≥7 or PDOP ≥8).

The proposed algorithm operates globally by calculating a kernel density surface based on the set of GPS points for each participant. It then extracts the peaks corresponding to the local density maxima, which become candidates for visited places. When staying at a given activity location, the recorded GPS locations tend to be normally distributed around a mean position that may be an acceptable approximation of the true location.

Because activity place detection is based on point density, the algorithm performs better when GPS points are sampled continuously. The interruption of the signal when people spend time inside a building does not result in the accumulation of GPS points at that place and would hinder the detection of the place by the algorithm. In order to address this concern, the algorithm fills the temporal gaps in the data with a simple linear interpolation prior to the estimation of the kernel density surface.

Based on kernel peak belonging rules, each GPS point is then either allocated to a detected place or not allocated to a place, i.e., belonging to a trip segment. Assessing the start and end points (and associated times) of each subset of points allocated to each identified place allows the algorithm to derive a list of all visits over the follow-up period to each detected place.

# Appendix 2: Application for the GPS-based prompted recall mobility survey

The project relied on a modified version of the Mobility Web Mapping (MWM) application initially developed in the BIXI project of Lise Gauvin.

**Appendix Figure 1 Screenshot of the modified version of the Mobility Web Mapping application that was used in the RECORD GPS Study (based on a fictive participant)**


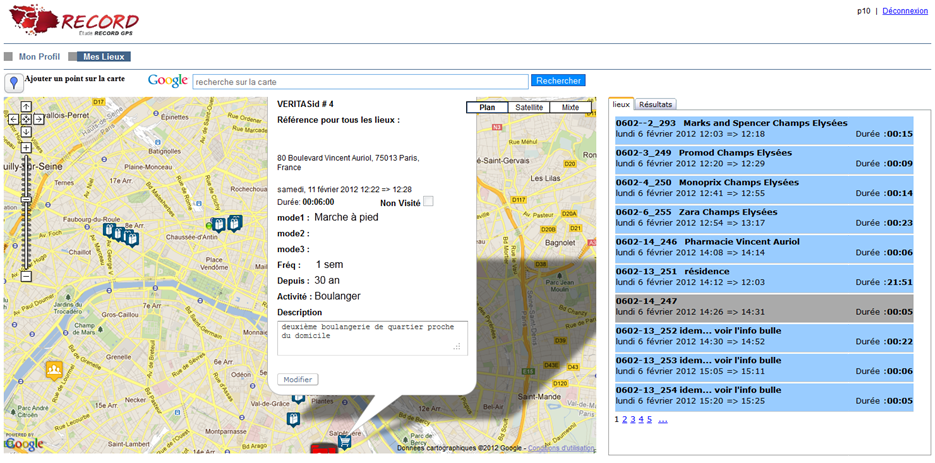


As illustrated in Appendix Figure 1, the main screen of this web application includes a panel (on the right) that reports in a chronological order all the visits to activity places identified by the algorithm, and a map where all the visits to activity locations appear. The GPS tracks themselves were not reported on the map.

For each visit to each place, the survey technician collected the following information (reported in a tooltip – i.e. a small window attached to the marker on the map opened by clicking on the marker or on the corresponding visit in the list of visits): type of activity practiced (typology of 34 options); the frequency of visit to that place per week, month, or year; the number of weeks, months, or years over which visits were made to that place; whether the place had been already visited over the 7-day measurement period, and if so its id code in the application (from a drop-down menu); whether the visited place was one of the regularly visited places identified with the VERITAS application and the VERITAS id of the place; and the transportation mode(s) that were used (typology of 19 options).

Indicating that a visited place had been previously visited over the follow-up and providing the id code of that previous visit allowed the survey technician to not have to fill again information on the activity type (unless a different activity was practiced than at the first visit) and on the time patterns of frequentation of the place. However, information on the transportation modes to arrive at the place had to be provided at each visit.

The survey technician had to report visits to activity places that were not detected by the algorithm, by geolocating the marker on the map and providing information on the place and visit, including the dates and hours of arrival and departure.

The survey technician could invalidate some of the automatically detected places in two types of circumstances: first if the participant had not been to that place or if the place detected did not correspond to a real activity place; and second if two immediately successive visits to the same place in fact corresponded to a unique visit to the same place. The latter case for example corresponds to an indoor static position of the participant where a distortion in the signal received by the GPS (e.g., reflection of the signal on another building) resulted in a spurious loop suggesting that the participant left the place and went back to it. In such circumstances, the survey technician had to use a specific answer field of the application to indicate that the second visit was in fact part of the immediately preceding visit to the place.

Given the time needed to prepare the survey, administer it, enter the corresponding data into the application, and perform the additional tasks of follow-up and given the important amount of information to collect (GPS-based transportation surveys often collect data only over one day), the survey technician could only survey one participant per day.

# Appendix 3: Verification of the mobility survey data

Mistakes in the 7-day survey data that the SAS program attempted to identify include: missing data for an activity place (name, activity type, frequency of visit, and transportation mode to arrive at the place); incoherence between the frequency of visit and the period over which visits were made to the place; incoherent assignment of a VERITAS regular destination to a visited place; incoherence of information provided for a place at two successive visits; more than one primary residence; the name of a supermarket does not include its brand; successive visits to the same place in fact corresponding to a unique visit do not have their start and end times comprised between the start and end times of the entire visit to the place; other incoherence in the declaration that a visited place was already visited over the previous days or in the aggregation of successive visits to the same place in a unique visit; visit to a place starting before the beginning or finishing after the end of the observation period; the survey data collection does not start at 0:00 am the first of the 7 days, i.e., the day after the recruitment, and/or does not end at 11:59 pm the seventh day; the end time of a visit occurred after its start time; and an overlap in the time spent at two different activity places. Moreover, an alert was generated when a participant was not at home, in an alternative residence, in a hotel, or at friends’ residences at 2:45 am or 3:15 am. As the activity place detection algorithm was ran on a daily basis (days from 3:00 am to 2:59 am the day after), a typical evening and night at home resulted in one visit at home until 2:59 am and one other visit at home from 3:00 am onward. The survey technician was asked to combine these two visits into one. An alert was also generated if the id of the place visited was not the same at 2:45 am and 3:15 am. Exceptions were introduced in the SAS program to ignore these alerts when relevant after checking with the survey technician.

*Length of follow-up*

The interruption of the follow-up before the end of the 7 days is a source of distortion in two different ways for the individual-level statistics computed by cumulating information over 7 days for each participant (trip-level analyses are largely unaffected by such incompleteness of the follow-up). Only few participants stopped the follow-up before the end of the study (often because of their mistake on the final day of the follow-up). In these cases, survey data on trips and activities were collected until the end of the 7-day period (in the absence of GPS and accelerometry data), except for certain participants (n = 8) for whom survey data were available only for 4 to 6 days. For these 8 participants, the impact of this first source of incompleteness (in survey data) on a number of individual-level statistics was compensated by applying weights inversely proportional to the percentage of the follow-up that was performed. More precisely, only taking into account the daytime period between 8:00 am and 10:00 pm over 7 days, a weight that was inversely proportional to the rate of coverage of this daytime period by the electronic mobility survey was applied to these statistics as a correction. Only the following statistics, which implied the accumulation of a quantity over 7 days, were corrected in this way: number of visits to activity places over 7 days; time spent in transportation; and cumulated accelerometry variables over 7 days (overall / in transportation).

Another potential source of incompleteness pertained to the accelerometry data (inappropriate periods of nonwear of the accelerometer over the 7 days during periods for which survey data are available). The assumption that periods of nonwear of the device detected from the accelerometry systematically corresponded to sleeping or resting time or to periods without moderate to vigorous physical activity (MVPA) is unlikely to hold. First, even if relatively rare, the non-recording of sport events that imply contact with the water would artificially increase the percentage of physical activity attributable to transportation. Missing accelerometry data for other sport or MVPA episodes performed at activity places would have a similar impact on this individual-level statistic. On the opposite, nonwear of the accelerometer during trips would spuriously decrease the percentage of physical activity and energy expenditure attributable to transportation. Overall, 7% of the trips identified in the final participants’ timetable over 7 days were found to overlap an episode of nonwear of the accelerometer. It was decided to not perform corrections for the individual-level statistics on the percentage of activity attributable to transportation because of a lack of information to do so: based on the timetable derived from the survey, physical activity values could have been imputed for trips with missing accelerometry data using information on the mode that was used; however, comparable information could not have been imputed for missing accelerometry data during time spent at activity locations, which information would be also needed to correct the percentage of physical activity made during transportation.

Differently, when simulating scenarios of shift of transportation modes, the available information on transportation modes was used to predict the number of minutes of MVPA for all the trips of the timetable that overlapped a period of nonwear of the accelerometer. The predicted physical activity values for these trips were used, except if they were lower than the observed values, to calculate the overall physical activity performed in the reference scenario (no change in the transportation modes).

# Appendix 4: Management of accelerometry data

Corrections were implemented for the participants for whom a daylight saving time change occurred during the collection period.

*Identification of nonwear of the accelerometer*

The following default settings of ActiLife 5.10 were used to identify episodes of nonwear of the accelerometer: floating windows of consecutive epochs with 3-axes counts equal to 0 for at least 60min with a Spike tolerance of 2min of nonzero epochs (ActiLife continues scoring a nonwear bout as nonwear until it detects more than the Spike Tolerance number of epochs above zero).

*Energy expenditure based on the Sasaki and Freedson formula*

The formula was provided in the article of Sasaki and Freedson . Five-second epochs were used. Axis1, axis2, and axis3 denote the counts for each of the 3 axes for 5-second epochs. The counts were rescaled on a 1 min basis:

VMCPM = sqrt(axis1*12*axis1*12 + axis2*12*axis2*12 + axis3*12*axis3*12)

CPM = axis1*12

The following formulas were then applied:

When VMCPM >2690:

METs = 0.000863*VMCPM + 0.668876

For the epochs of 5 seconds, the energy expenditure is (weight = p kg; MET = X):

EE = X × p / 720 kcal

When VMCPM ≤2690: (use of the Work-Energy theorem)

EE_kcals = CPM*0.0000191*weight

The approach from the Actigraph website was followed here: the Work-Energy theorem based on uniaxial acceleration was used to fill the range of values for which the newer equation did not apply: VMCPM ≤2690.

*Energy expenditure based on the formula provided on the Actigraph website*

The formula was taken from the Actigraph website . Five-second epochs were used. Axis1, axis2, and axis3 denote the counts for each of the 3 axes for 5-second epochs. The counts were rescaled on a 1 min basis:

VMCPM = sqrt(axis1*12*axis1*12 + axis2*12*axis2*12 + axis3*12*axis3*12)

CPM = axis1*12

When VMCPM >2453:

EE_kcals = 0.001064*VMCPM + 0.087512*weight – 5.500229

When VMCPM ≤2453: (use of the Work-Energy theorem)

EE_kcals = CPM*0.0000191*weight

To retrieve the information for the 5-second epochs:

EE_kcals_fin = EE_kcals / 12

*Energy expenditure from the refined Crouter equation*

The refined Crouter equation is based on axis 1 counts only . The ActiLife software provides METs estimated from the Crouter algorithm for epochs of 10 seconds.

For a MET of X, for an epoch of 10 seconds, and for a person of weight p (in kg) (as derived from the following formula: EE = X × p kcal∙h^-1^ = X × p / 60 kcal∙min^-1^):

EE = X × p / 360 kcal

*Moderate to vigorous physical activity*

Each 5-second epoch was classified as with MVPA or not. The cutoff provided by Sasaki and Freedson was used: >2690 counts min^–1^ on the 3 axes. In the present study, each 5-second epoch was classified as with MVPA if the 3-axes count was above 2690/12.

*Sedentary time*

Sedentary time was first assessed for each 5-second epoch. For each 5-second epoch, the vector magnitude based on the 3 axes was rescaled on a 1 min scale:

VMCPM = sqrt(axis1*12*axis1*12 + axis2*12*axis2*12 + axis3*12*axis3*12)

Each epoch was classified as sedentary if VMCPM <150 .

Sedentary time was also assessed on a 1 min basis. The 3-axes vector magnitude counts provided by ActiLife were aggregated on a 1 min basis. Each min was classified as sedentary if the aggregated vector magnitude counts were >150.

*French recommendation for physical activity*

The current French recommendation for physical activity is of at least the equivalent of 30min of brisk walk per day (as an activity of moderate intensity), or alternatively the equivalent of at least 20min per day of physical activities of vigorous intensity.

For the examination of scenarios of shift of transportation modes in the present study, as a simplification, the recommendation of at least 30min of MVPA per day was retained. It would have required additional calculation rules to distinguish between activities of moderate intensities and activities of vigorous intensities in the calculation of whether each participant achieved the recommendation.

# Appendix 5: Analyses with the accelerometry low frequency extension filter activated

As a sensitivity analysis, the analyses were repeated with the accelerometry low frequency extension filter activated. The regression models provided in Appendix Table 1, with the low frequency extension filter activated, can be compared with the models provided in Table 3 of the main article, with the normal filter. The findings were comparable with the two approaches.

| **Appendix Table 1 Trip-level associations between the transportation mode used and physical activity and energy expenditure (time-standardized outcomes, low frequency extension filter) (n = 6,164 or 5,867 trips, N = 234 participants)^a^** | | | | |
| --- | --- | --- | --- | --- |
| **Transportation mode variable** | **Number of steps taken per 10min of trip**  **β (95% CI)** | **MVPA per 10min of trip (min)**  **β (95% CI)** | **Sedentary time per 10min of trip (min)^b^**  **β (95% CI)** | **Energy expenditure per 10min of trip (kcal)^c^**  **β (95% CI)** |
| **Crude classification** |  |  |  |  |
| Personal motorized vehicle | Ref. | Ref. | Ref. | Ref. |
| Public transportation | 135.7 (111.5, 160.0) | 1.9 (1.6, 2.1) | –0.0 (–0.2, 0.2) | 11.0 (9.6, 12.5) |
| Biking | 237.9 (189.7, 286.0) | 0.5 (0.1, 1.0) | –2.6 (–3.0, –2.3) | 6.5 (3.7, 9.3) |
| Walking | 408.4 (390.6, 426.3) | 4.5 (4.4, 4.7) | –2.6 (–2.8, –2.5) | 26.7 (25.7, 27.8) |
|  |  |  |  |  |
| **Detailed classification** |  |  |  |  |
| 4-wheel motor, driving | Ref. | Ref. | Ref. | Ref. |
| 4-wheel motor, passenger | 13.5 (–31.4, 58.3) | 0.0 (–0.4, 0.4) | 0.2 (–0.2, 0.5) | 1.0 (–1.6, 3.6) |
| 2-wheel motor vehicle | 123.1 (52.8, 193.4) | –0.2 (–0.8, 0.5) | –1.8 (–2.3, –1.2) | –1.5 (–5.6, 2.7) |
| Metro | 148.5 (112.8, 184.2) | 2.0 (1.7, 2.4) | –0.3 (–0.6, –0.1) | 11.8 (9.8, 13.9) |
| Bus / coach | 168.0 (119.7, 216.2) | 1.7 (1.2, 2.1) | –0.7 (–1.1, –0.3) | 10.7 (7.9, 13.5) |
| Train | 155.7 (100.5, 211.0) | 2.0 (1.5, 2.5) | –0.1 (–0.4, 0.5) | 12.6 (9.4, 15.8) |
| Tramway | 254.0 (130.5, 377.6) | 3.0 (1.8, 4.1) | –0.9 (–1.9, 0.1) | 15.9 (8.8, 22.9) |
| Biking | 242.6 (192.9, 292.3) | 0.5 (0.0, 1.0) | –2.7 (–3.1, –2.3) | 6.5 (3.6, 9.4) |
| Walking | 417.8 (398.3, 437.2) | 4.5 (4.3, 4.7) | –2.7 (–2.9, –2.6) | 26.8 (25.7, 28.0) |
| CI = confidence interval; MVPA = moderate to vigorous physical activity.  ^a^The multilevel linear models included a random effect at the individual level, and were not adjusted for any other covariate. The crude and the detailed transportation mode variables were introduced in separate models.  ^b^Each 5 second epoch was classified as sedentary or not (the regression coefficients were *a posteriori* converted in min of sedentary time).  ^c^Energy expenditure was calculated according to the formula of Sasaki and Freedson. | | | | |

The only differences that were apparent are that, when the low frequency extension filter was activated:

(i) the difference in the number of steps taken between biking trips and trips with a personal motorized vehicle was much larger;

(ii) the difference in the number of steps taken between public transportation trips and trips with a personal motorized vehicle was less important;

(iii) differences in sedentary time between, on the one hand, walking, biking, or public transportation trips, and on the other hand, trips with a personal motorized vehicle were less important.

Appendix Table 2 provides the percentage of accelerometry that was attributable to transportation when the low frequency extension filter for accelerometry was activated.

| **Appendix Table 2 Percentage of the accelerometry-derived physical activity, energy expenditure, and sedentary time attributable to transportation when the low frequency extension filter was activated** | | |
| --- | --- | --- |
|  | **Median** | **Interdecile range** |
| Number of steps taken | 33% | 17% – 48% |
| Energy expenditure (Actigraph) | 30% | 11% –48% |
| Energy expenditure (Sasaki) | 30% | 11% – 48% |
| Energy expenditure (Crouter) | 20% | 10% – 30% |
| Moderate to vigorous physical activity | 31% | 11% – 50% |
| Sedentary time (epoch based definition) | 12% | 5% – 22% |
| Sedentary time (minute based definition) | 12% | 5% – 23% |

# Appendix 6: Definition of the samples of trips in which each analysis was performed and related sample sizes

| **Appendix Table 3 Definition of the samples of trips in which each analysis was performed and related sample sizes** | | |
| --- | --- | --- |
| Statistics / analyses | **Definition of the sample** | **Sample size (trips)** |
| Percentage of trips overlapping a period of nonwear of the accelerometer | Full sample of trips | 7,650 |
| Median street network distance of trips; median time length of trips | Exclusion of trips starting before the 7-day period (incomplete trips) | 7,644 |
| Proportion of trips with each mode | Additional exclusion of trips with two or more nonwalking modes; and of trips with another transportation mode than in the classification employed | 7,468 |
| Relationships between transportation modes and physical activity (larger sample) | Additional exclusion of: trips of less than 1 min (appearing with a 0 min length in the database); trips that overlapped a period of nonwear of the accelerometer; trips of less than 5m of length; trips that started and ended at the same location; recreational walking trips or atypical trips; trips with an excessive duration compared with the distance covered and the mode used; and trips starting and/or ending out of the Ile-de-France region | 6,164 and 5,867 |
| Relationships between transportation modes and physical activity (more accurate data) | Additional exclusion of trips for which the start and/or end times were edited or generated during the survey | 3,423 and 3,249 |

# Appendix 7: Exclusion of trips of an excessive length

Cutoff values of duration reported in Appendix Table 4 were used to identify trips of an excessive duration compared with the street network distance of the trip, for each transportation mode. For example, trips between 1 and 2km of length were considered atypical if the duration of the trip was greater than 105min for a walking trip or greater than 90min for a trip by public transportation.

Such atypical trips were excluded from the modeling of associations between transportation modes and physical activity for a proper calibration of the models. These atypical trips were not eligible to changes of transportation modes in the scenarios of shift of modes: the observed MVPA time for these atypical trips was directly used in the calculation of the cumulated MVPA time both in the reference situation and in the two scenarios of shift.

In addition to the trips excluded from the modeling with the criteria in Appendix Table 4, all trips within the Ile-de-France region of more than 240min were defined as atypical.

| **Appendix Table 4 Definition of trips with an excessive duration, according to the (shortest) street network distance and mode used** | | | | | |
| --- | --- | --- | --- | --- | --- |
| **Length of the trip in km** |  | **Maximum duration of the trip in min with the following mode:** | | | |
|  |  | **Public transportation** | **Personal motorized vehicle** | **Bike** | **Walking** |
| 0–1 |  | 75 | 75 | 75 | 75 |
| 1–2 | 90 | | 90 | 90 | 105 |
| 2–3 | 105 | | 105 | 105 | 120 |
| 3–5 | 150 | | 120 | 120 | 180 |
| 5–10 | 180 | | 150 | 150 | 240 |
| 10–50 | 240 | | 210 | 240 |  |
|  |  | |  |  |  |

# Appendix 8: Models of relationships between transportation modes and physical activity adjusted for trip-level and individual-level variables

*Should the main association of interest be adjusted for trip-level and individual-level variables?*

The directed acyclic graphs represented in Appendix Figures 2A and 2B show that confounding was not expected in the main relationship of interest, between the transportation mode employed in a given trip and the physical activity and energy expenditure during that trip (represented in red in Appendix Figure 2B). In Appendix Figure 2A focusing on the relationship at the individual level, the individual demographic and socioeconomic variables likely influence: (i) the type of transportation modes used in daily trips; (ii) the mobility habits of people and frequency of trips; and (iii) the engagement in the different other forms of physical activity. Due to the last two associations, individual sociodemographic variables confound the relationship between the transportation modes that are used and the overall physical activity of the participants. The individual-level relationship between the transportation modes that are used and transportation-related physical activity is also confounded, e.g., by the frequency of trips and patterns of mobility. However, the directed acyclic graph at the trip level in Appendix Figure 2B suggests that there is no particular reason to expect confounding in the relationship between the transportation modes used in a given trip and the physical activity made in the corresponding trip.

In the present study, transportation is a well-defined activity, with information on the start and end times of each trip, which excludes the practice of activity at given places. There is relatively little room for being more or less active when traveling with a specific mode (during the trip stage entirely devoted to transportation with a mode), so differences in physical activity levels for 10min or 1km of trip associated with a trip characteristic or an individual sociodemographic characteristic would likely be related to the fact that certain types of trips (e.g., longer ones, those made on the week-end, those made in the suburb) or the trips of certain sociodemographic groups (e.g., of males, elders, low educated individuals) comprise a larger or smaller proportion of the trip distance or length made by active modes (e.g., walking). The assumption is that the individual sociodemographic and trip-level variables are associated with the accelerometry of a given trip only through their influence on the share of active transportation in the trip. It suggests that there is no causal effect of these individual-level or trip-level variables independent of the path through the transportation modes, and therefore that there is no confounding .

Overall, the purpose of including trip-level variables or individual sociodemographic variables in the regression models was not to remove confounding, but was to identify variations in the magnitude of the relationship between transportation modes and physical activity between types of trips or types of individual sociodemographic groups.

**Appendix Figure 2A Directed acyclic graph at the individual level on the relationships between the transportation modes used and physical activity**


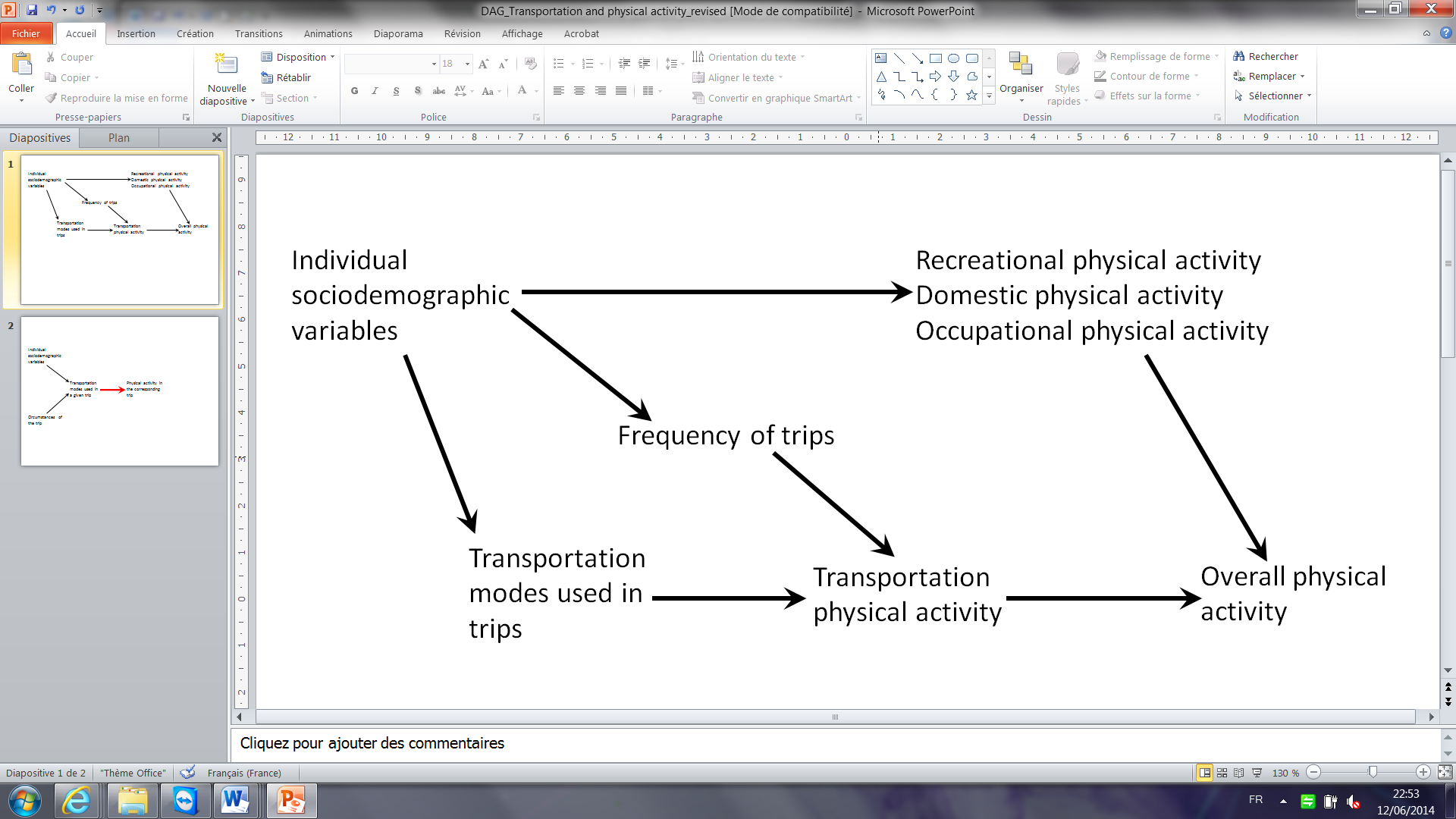


**Appendix Figure 2B Directed acyclic graph at the trip level on the relationships between the transportation modes used and physical activity**


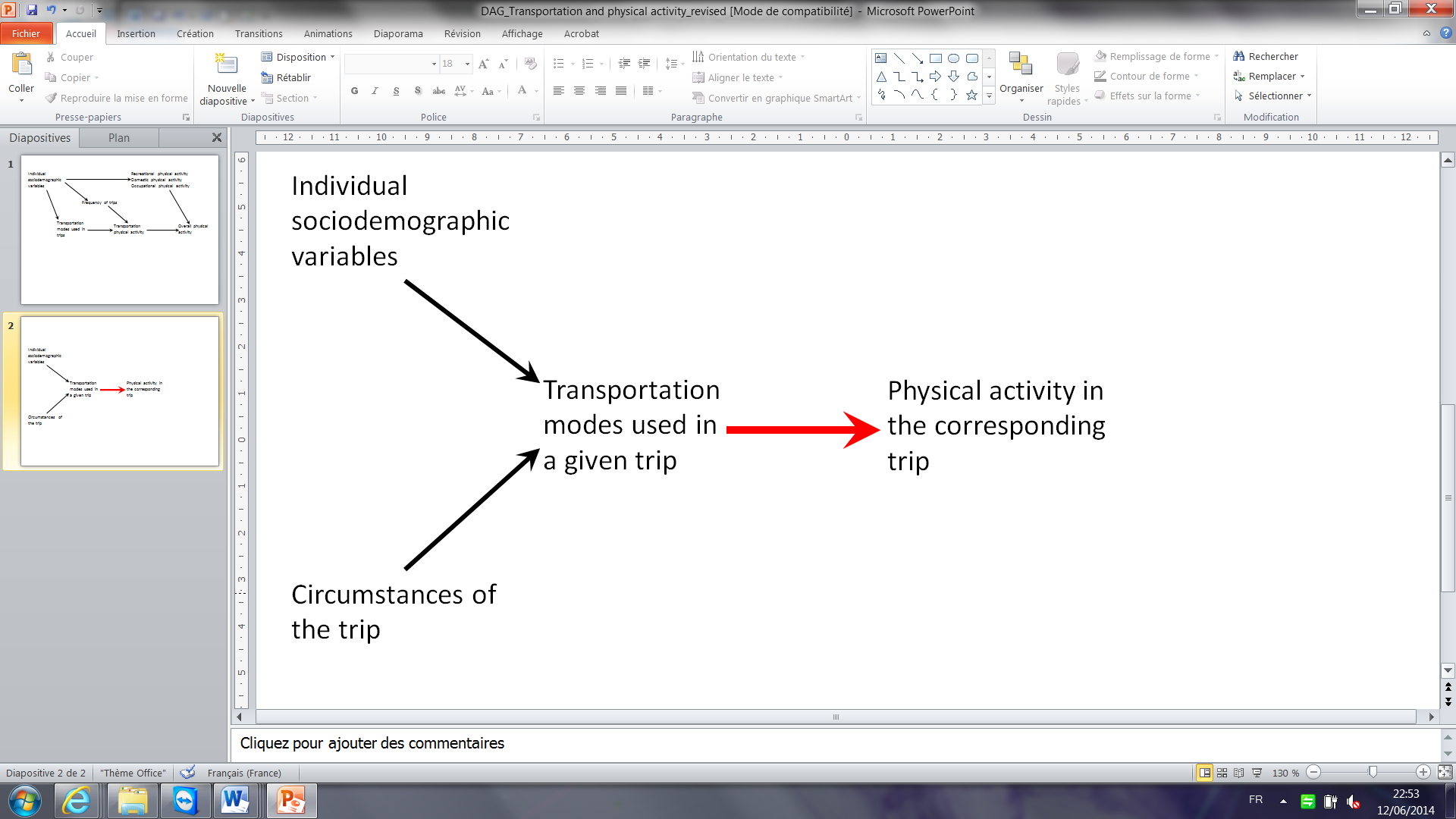


*Incorporation of individual-level and trip-level variables in the analyses*

Apart from the estimated distance covered during the trip and the duration of the trip, the other trip-level variables considered included the hour of departure of the trip (morning: 5:00 am to 9:59 am; day: 10:00 am to 4:59 pm; evening: 5:00 pm to 11:59 pm; and night: 0:00 am to 4:59 am) and the day of departure of the trip (week or weekend day). Additionally, age (35–49; 50–64; and 65 years or over) and gender were considered.

These variables were introduced as main effects in the models and as interacting with the transportation mode variable (crudest classification). These parameters allowed us to assess, respectively: (i) whether specific trips or trips of specific individuals were associated with an increased physical activity, uniformly for all transportation modes; or (ii) whether differences between transportation modes in the physical activity accumulated during the trip varied in magnitude according to the type of trip or to the type of individual.

The distance and the duration of the trip were considered as main effects and modifiers of the effect of the transportation mode, in addition to their use in the standardization of the accelerometry variables. The standardization of accelerometry variables by distance or time permits to account for the fact that a longer trip is on average associated with a larger amount of physical activity. Differently, accounting for the distance or time length of trips as a main effect and interaction terms (with transportation modes) allowed us to account for the fact that the amount of physical activity per unit of time or distance may vary with the overall duration or distance of the trip, uniformly for all modes (main effect) or specifically for certain modes (interaction).

Interactions of effects were systematically tested between each trip-level or individual-level variable and the transportation mode variable. Interactions were retained only if the associated p-value was <0.01.

Appendix Table 5 reports the regression models for the accelerometry outcomes considered in the main article, while Appendix Table 6 provides the corresponding regression models for the complementary outcomes. All such regression models were estimated with time-standardized variables.

All the regression models of Appendix Tables 5 and 6 are not systematically commented here. As an example, the model for time-standardized MVPA is briefly described (Appendix Table 5). In this model, an increase in the duration of the trip was associated with a decrease in the excess time of MVPA observed for walking or public transportation trips compared to trips with a personal motorized mode. This is likely due to the fact that longer walking trips may be at a lower speed or imply short stops over the way. For public transportation trips, it may also be attributable to the fact that in longer trips, the walk segments at the beginning and at the end of the trip account for a smaller proportion of the trip.

On the opposite, an interaction between the distance covered during the trip and walking suggests that a larger difference in MVPA time per 10min of trip between walking trips and trips with a personal motorized vehicle was observed in longer trips than in shorter trips. It may be attributable to the fact that longer walking trips imply a higher speed of walk, while longer car trips imply a smaller proportion of the trip devoted to walk to or from the vehicle.

It is more difficult to understand why differences in MVPA time for each 10min of trip between walking or biking trips and trips with a personal motorized vehicle were larger among men than women, after adjustment for the duration of the trip and the distance covered during the trip. Potential explanations include the imperfect adjustment for the distance covered (recalculated street network distance rather than effective distance), which imperfect adjustment could hide a higher speed of walk or cycling among men.

| **Appendix Table 5 Trip-level associations between, on the one hand, transportation modes, trip-level variables, and individual sociodemographic variables and, on the other hand, physical activity and energy expenditure (time-standardised variables) (n = 6,164 trips, N = 234 participants)^a^** | | | | |
| --- | --- | --- | --- | --- |
|  | **Number of steps taken per 10min of trip**  **β (95% CI)** | **MVPA per 10min of trip (min)**  **β (95% CI)** | **Sedentary time per 10min of trip (min)^b^**  **β (95% CI)** | **Energy expenditure per 10min of trip (kcal)^c^**  **β (95% CI)** |
| Transportation mode (vs. personal motorized vehicle) |  |  |  |  |
| Public transportation | 268.0 (225.7, 310.3) | 2.3 (1.9, 2.6) | –0.8 (–1.0, –0.6) | 10.9 (9.5, 12.3) |
| Biking | –26.2 (–104.0, 51.6) | –0.5 (–1.2, 0.2) | –2.5 (–2.9, –2.1) | 3.8 (1.2, 6.5) |
| Walking | 453.1 (415.4, 490.7) | 3.6 (3.3, 3.9) | –2.5 (–2.8, –2.3) | 18.0 (16.3, 19.7) |
| Trip length (10min units) | 3.6 (–2.5, 9.7) | 0.1 (–0.0, 0.1) | 0.0 (–0.0, 0.1) | –0.0 (–0.3, 0.2) |
| × walking | –64.7 (–75.2, –54.2) | –0.6 (–0.7, –0.5) | 0.3 (0.2, 0.4) | –3.1 (–3.6, –2.6) |
| × public transportation | –13.6 (–22.0, –5.2) | –0.1 (–0.2, –0.0) |  |  |
| Distance covered (in km) | –4.1 (–5.5, –2.8) | –0.0 (–0.1, –0.0) | 0.1 (0.1, 0.1) | –0.2 (–0.3, –0.2) |
| × walking | 125.1 (106.8, 143.4) | 1.0 (0.9, 1.2) | –0.7 (–0.9, –0.6) | 6.4 (5.5, 7.4) |
| Hour of departure (vs. morning) |  |  |  |  |
| Day | –8.6 (–32.9, 15.8) | –0.2 (–0.4, –0.0) | 0.2 (–0.0, 0.3) | –1.1 (–2.2, 0.0) |
| × walking | –41.5 (–71.8, –11.3) |  |  |  |
| Evening | –33.3 (–55.6, –11.0) | –0.3 (–0.5, –0.1) | 0.3 (0.1, 0.5) | –1.7 (–2.9, –0.6) |
| Night | –42.6 (–137.4, 52.1) | –0.2 (–1.1, 0.6) | 0.7 (–0.1, 1.4) | –0.9 (–5.9, 4.1) |
| Weekend (vs. week) | 35.8 (18.7, 52.9) | 0.3 (0.2, 0.5) | –0.4 (–0.5, –0.3) | 2.0 (1.1, 2.9) |
| Age (in years) |  |  |  | –0.1 (–0.2, –0.1) |
| Male (vs. female) | –18.3 (–52.8, 16.2) | –0.0 (–0.4, 0.3) | 0.3 (0.0, 0.6) | 2.8 (1.0, 4.7) |
| × walking | 64.7 (30.8, 98.6) | 0.7 (0.4, 1.0) | –0.5 (–0.8, –0.3) | 8.1 (6.3, 9.8) |
| × biking | 164.6 (65.3, 263.9) | 1.2 (0.3, 2.0) |  |  |
| CI = confidence interval; MVPA = moderate to vigorous physical activity.  ^a^The multilevel linear models included a random effect at the individual level. The crude transportation mode variable was used. Time-standardized outcomes were used.  ^b^Each 5 second epoch was classified as sedentary or not (the regression coefficients were *a posteriori* converted in min of sedentary time).  ^c^Energy expenditure was calculated according to the formula of Sasaki and Freedson. | | | | |

| **Appendix Table 6 Trip-level associations between, on the one hand, transportation modes, trip-level variables, and individual sociodemographic variables and, on the other hand, physical activity and energy expenditure (complementary outcomes, time-standardised variables) (n = 6,164 trips, N = 234 participants)^a^** | | | |
| --- | --- | --- | --- |
|  | **Sedentary time per 10min of trip (min)^b^**  **β (95% CI)** | **Energy expenditure per 10min of trip (kcal)^c^**  **β (95% CI)** | **Energy expenditure per 10min of trip (kcal)^d^**  **β (95% CI)** |
| Transportation mode (vs. personal motorized vehicle) |  |  |  |
| Public transportation | –0.3 (–0.7, 0.2) | 11.2 (9.8, 12.7) | 8.1 (6.9, 9.3) |
| Biking | –2.7 (–3.2, –2.1) | 4.2 (1.5, 6.9) | 8.6 (6.4, 10.7) |
| Walking | –0.4 (–1.3, 0.4) | 18.2 (16.5, 20.0) | 15.3 (13.9, 16.7) |
| Trip length (10min units) |  | –0.0 (–0.3, 0.3) | –0.1 (–0.3, 0.1) |
| × walking |  | –3.3 (–3.7, –2.6) | –2.2 (–2.7, –1.8) |
| Distance covered (in km) | 0.1 (0.1, 0.1) | –0.3 (–0.2, –0.2) | –0.3 (–0.3, –0.2) |
| × walking | –0.4 (–0.5, –0.2) | 6.5 (5.6, 7.5) | 3.2 (2.4, 4.0) |
| Hour of departure (vs. morning) |  |  |  |
| Day | –0.0 (–0.3, 0.3) | –1.1 (–2.2, 0.1) | –0.4 (–1.2, 0.6) |
| × walking | 0.5 (0.2, 0.8) |  |  |
| Evening | 0.4 (0.2, 0.6) | –1.8 (–3.0, –0.6) | –1.3 (–2.3, –0.4) |
| Night | 1.0 (0.0, 2.0) | –1.0 (–6.1, 4.1) | –1.9 (–5.9, 2.2) |
| Weekend (vs. week) | –0.3 (–0.5, –0.1) | 2.1 (1.2, 3.0) | 1.9 (1.2, 2.7) |
| Age (in years) | 0.0 (–0.0, 0.0) | –0.1 (–0.2, –0.1) | –0.2 (–0.2, –0.1) |
| × walking | –0.0 (–0.0, –0.0) |  |  |
| Male (vs. female) | 0.8 (0.4, 1.2) | 3.3 (1.4, 5.3) | 4.6 (2.9, 6.4) |
| × walking | –1.1 (–1.5, –0.7) | 8.9 (7.1, 10.7) | 5.3 (3.8, 6.7) |
| × public transportation | –0.7 (–1.3, –0.2) |  |  |
| CI = confidence interval.  ^a^The multilevel linear models included a random effect at the individual level. The crude transportation mode variable was used. Time-standardized outcomes were used.  ^b^Each min was classified as sedentary or not.  ^c^Energy expenditure was calculated according to the Actigraph formula.  ^d^Energy expenditure was calculated according to the Crouter 2-regression model. | | | |

# Appendix 9: Justifications for the values of parameters to determine the probability of change of mode in each trip in the simulations

Parameters had to be chosen to calculate the probability of change of mode in a trip. This probability of shift was equal to the basal probability from the scenario, plus an individual term, plus a penalty term. As noted in the main text, the individual term was a random value between –0.1 and +0.1 on the probability scale. It permits to take into account that participants changing of mode for one of their trips would have a greater likelihood to make a comparable change for their other trips. This parameter allows the simulations to distinguish between individuals who would be for some reason sensitive to the intervention and individuals less sensitive to it. Considering the basal probabilities of shift according to the length of the trip and the mode reported in Table 1 of the main article, the individual term was calibrated to imply substantial between-individual variations (20 percentage points at the most) but to correspond to variations in the probability of change of smaller magnitude than that related to the length of the trip itself.

For shifts from personal motorized vehicle to public transportation, a penalty of 0.05, 0.1, 0.1, and 0.2 on the probability scale was applied, respectively, to trips between Paris and the suburb/provinces; trips from the suburb/provinces to the suburb/provinces; trips on a Sunday; and trips between 0:00 am and 5:00 am. The first two parameters were chosen to reflect the highly centralized nature of the public transportation system in the Ile-de-France region where the density of the network decreases with increasing distance from Paris and where it is often difficult or even impossible to use public transportation to travel from one suburban location to another suburban location. The last two parameters were intended to reflect the fact that traveling on a Sunday and traveling during the night represent obstacles of comparable or still greater magnitude, as a large number of public transportation lines do not work on Sunday and most of them are closed during the night.

# Appendix 10: Percentage of physical activity attributable to transportation

*Percentage of physical activity attributable to transportation for the alternative outcomes*

Based on the definition available on the Actigraph website , the percentage of energy expenditure that was attributable to transportation was 30% (interdecile range: 12%, 50%). Based on the Crouter definition , the corresponding percentage was of 20% (interdecile range: 10%, 30%). The sharp difference in the percentage of energy expended attributable to transportation between the Crouter definition and the other two definitions is related to the fact that for epochs with a 0 count on each of the three axes, the Crouter formula attributes a MET of 1 and a nonzero energy expenditure while the two other definitions attribute an energy expenditure of 0 kcal. Epochs with a vector magnitude equal to 0 are much more frequent during periods at activity places than during trips, leading to differences in the percentage of energy expended attributable to transportation with the different definitions.

When sedentary time was assessed on a min basis, the percentage of sedentary time attributable to transportation was of 13% (interdecile range: 5%, 25%).

*Partial correlations between the percentage of activity attributable to transportation and activity levels during transportation or at activity places*

The aim was to assess whether variations between individuals in the percentage of physical activity made during transportation were most strongly associated with the overall activity level during transportation or with the overall activity level at activity places. Partial correlations allow one to adjust the estimate of a correlation for a given factor.

Adjusted for the number of steps at activity places, the correlation between the percentage of steps attributable to transportation and the number of steps made during transportation was 0.91. Adjusted for the number of steps during transportation, the correlation between the percentage of steps attributable to transportation and the number of steps made at activity places was –0.79.

Adjusted for energy expenditure (Sasaki and Freedson definition) at activity places, the correlation between the percentage of energy expenditure attributable to transportation and energy expenditure during transportation was 0.91. Adjusted for energy expenditure during transportation, the correlation between the percentage of energy expenditure attributable to transportation and energy expenditure at activity places was –0.81.

Adjusted for MVPA at activity places, the correlation between the percentage of MVPA attributable to transportation and MVPA made during transportation was 0.91. Adjusted for MVPA during transportation, the correlation between the percentage of MVPA attributable to transportation and MVPA made at activity places was –0.80.

Overall, these adjusted correlations suggest that variations between participants in the overall physical activity made during trips and variations between participants in the overall physical activity made at activity places contributed to a comparable extent to the observed between-participant variations in the percentage of activity devoted to transportation. It should be noted that the point estimate of the correlations was slightly larger with physical activity made during trips, suggesting that variations in the overall activity made during transportation contributed at least as much than variations in physical activity at activity places.

# Appendix 11: **Additional models estimated for the accelerometry outcomes**

The following pages report additional models that were estimated between the transportation mode variable and the accelerometry outcomes:

- in Appendix Table 7: models for the complementary outcomes (sedentary time determined on a min basis and energy expenditure based on the Actigraph definition and on the Crouter definition ), with unstandardized outcomes;

- in Appendix Table 8: models for the complementary outcomes with time-standardized or distance-standardized outcomes;

- in Appendix Table 9: models for the four main outcomes (time-standardized variables), after excluding from the modeling the trips whose start or end times were edited or reported during the survey, as opposed to trips whose start or end times were identified by the automatic algorithm and confirmed during the survey;

- in Appendix Table 10: models for the four main outcomes (distance-standardized variables), after excluding from the modeling the trips whose start or end times were edited or reported during the survey, as opposed to trips whose start or end times were identified by the automatic algorithm and confirmed during the survey.

| **Appendix Table 7 Trip-level associations between the transportation mode used and physical activity and energy expenditure (complementary outcomes, unstandardised variables) (n = 6,164 or 5,867 trips, N = 234 participants)^a^** | | | |
| --- | --- | --- | --- |
| **Transportation mode variable** | **Sedentary time (min)^b^**  **β (95% CI)** | **Energy expenditure (kcal)^c^**  **β (95% CI)** | **Energy expenditure (kcal)^d^**  **β (95% CI)** |
| **Crude classification** |  |  |  |
| Personal motorized vehicle | Ref. | Ref. | Ref. |
| Public transportation | 8.8 (7.8, 9.8) | 62.9 (59.0, 66.8) | 87.1 (81.7, 92.5) |
| Biking | –10.3 (–12.3, –8.3) | 12.0 (4.2, 19.7) | 16.5 (5.9, 27.2) |
| Walking | –10.0 (–10.7, –9.2) | 23.4 (20.5, 26.2) | –2.1 (–6.0, 1.9) |
|  |  |  |  |
| **Detailed classification** |  |  |  |
| 4-wheel motor, driving | Ref. | Ref. | Ref. |
| 4-wheel motor, passenger | 2.8 (1.1, 4.6) | 0.2 (–6.7, 7.2) | 3.7 (–5.5, 12.9) |
| 2-wheel motor vehicle | –7.2 (–9.9, –4.5) | 8.1 (–2.8, 19.1) | 9.9 (–4.6, 24.4) |
| Metro | 3.1 (1.7, 4.5) | 54.2 (48.7, 59.7) | 69.6 (62.2, 76.9) |
| Bus / coach | 2.3 (0.5, 4.2) | 39.0 (31.6, 46.4) | 51.8 (41.9, 61.7) |
| Train | 10.3 (8.2, 12.4) | 81.6 (73.1, 90.1) | 101.2 (89.8, 112.5) |
| Tramway | –0.5 (–5.3, 4.2) | 36.6 (17.7, 55.6) | 28.6 (3.3, 53.8) |
| Biking | –10.2 (–12.1, –8.3) | 13.0 (5.3, 20.7) | 18.6 (8.4, 28.8) |
| Walking | –10.5 (–11.2, –9.7) | 23.1 (20.0, 26.1) | –2.6 (–6.6, 1.4) |
| CI = confidence interval.  ^a^The multilevel linear models were not adjusted for any other covariate. The crude and the detailed transportation mode variables were introduced in separate models.  ^b^Each min was classified as sedentary or not.  ^c^Energy expenditure was calculated according to the Actigraph formula.  ^d^Energy expenditure was calculated according to the Crouter 2-regression model. | | | |

| **Appendix Table 8 Trip-level associations between the transportation mode used and physical activity and energy expenditure (complementary outcomes, time-standardized and distance-standardized variables) (n = 6,164 or 5,867 trips, N = 234 participants)^a^** | | | |
| --- | --- | --- | --- |
| **Transportation mode variable** | **Sedentary time per 10min or km of trip (min)^b^**  **β (95% CI)** | **Energy expenditure per 10min or km of trip (kcal)^c^**  **β (95% CI)** | **Energy expenditure per 10min or km of trip (kcal)^d^**  **β (95% CI)** |
| **Time-standardized outcomes** |  |  |  |
| **Crude classification** |  |  |  |
| Personal motorized vehicle | Ref. | Ref. | Ref. |
| Public transportation | –0.6 (–0.8, –0.3) | 11.1 (9.7, 12.6) | 7.5 (6.4, 8.6) |
| Biking | –3.3 (–3.8, –2.8) | 6.0 (3.2, 8.8) | 10.2 (8.0, 12.4) |
| Walking | –3.0 (–3.1, –2.8) | 26.5 (25.5, 27.6) | 20.0 (19.2, 20.9) |
|  |  |  |  |
| **Detailed classification** |  |  |  |
| 4-wheel motor, driving | Ref. | Ref. | Ref. |
| 4-wheel motor, passenger | 0.2 (–0.3, 0.7) | 1.2 (–1.4, 3.8) | 0.4 (–1.6, 2.5) |
| 2-wheel motor vehicle | –2.7 (–3.4, –1.9) | –1.8 (–5.9, 2.3) | 1.2 (–2.1, 4.5) |
| Metro | –1.1 (–1.4, –0.7) | 11.9 (9.8, 14.0) | 8.6 (7.0, 10.3) |
| Bus / coach | –1.0 (–1.5, –0.5) | 10.7 (70.9, 13.5) | 8.2 (6.0, 10.5) |
| Train | –0.6 (–1.2, –0.1) | 12.7 (9.5, 15.9) | 8.0 (5.5, 10.6) |
| Tramway | –1.5 (–2.8, –0.2) | 15.8 (8.7, 22.9) | 10.4 (4.8, 16.0) |
| Biking | –3.4 (–3.9, –2.8) | 6.0 (3.1, 8.9) | 10.3 (8.0, 12.6) |
| Walking | –3.1 (–3.3, –2.9) | 26.6 (25.5, 27.8) | 20.3 (19.4, 21.2) |
|  |  |  |  |
| **Distance-standardized outcomes** |  |  |  |
| **Crude classification** |  |  |  |
| Personal motorized vehicle | Ref. | Ref. | Ref. |
| Public transportation | 0.5 (1.1, 2.1) | 8.1 (0.2, 16.0) | 6.9 (–3.7, 17.4) |
| Biking | –1.5 (–4.7, 1.7) | 4.6 (–11.1, 20.4) | 10.8 (–10.3, 31.9) |
| Walking | 4.1 (2.9, 5.2) | 60.5 (54.7, 66.4) | 80.1 (72.3, 88.0) |
|  |  |  |  |
| **Detailed classification** |  |  |  |
| 4-wheel motor, driving | Ref. | Ref. | Ref. |
| 4-wheel motor, passenger | 0.7 (–2.4, 3.7) | 4.7 (–10.2, 19.5) | 7.3 (–12.7, 27.2) |
| 2-wheel motor vehicle | –3.0 (–7.5, 1.5) | –38.1 (–61.0, –15.1) | –26.3 (–56.6, 4.1) |
| Metro | 0.6 (–1.8, 3.0) | 8.4 (–3.4, 20.2) | 9.7 (–6.1, 25.6) |
| Bus / coach | 2.5 (–0.8, 5.9) | 17.2 (1.1, 33.3) | 24.4 (2.7, 46.2) |
| Train | –1.3 (–5.0, 2.5) | 2.9 (–15.5, 21.2) | –5.1 (–29.8, 19.6) |
| Tramway | 0.0 (–8.6, 8.7) | 4.8 (–36.7, 46.2) | 5.1 (–51.2, 61.5) |
| Biking | –1.6 (–4.8, 1.7) | 3.7 (–12.6, 20.1) | 10.7 (–11.2, 32.5) |
| Walking | 4.0 (2.7, 5.3) | 59.3 (53.0, 65.7) | 79.9 (71.4, 88.4) |
| CI = confidence interval.  ^a^The multilevel linear models were not adjusted for any other covariate. The crude and the detailed transportation mode variables were introduced in separate models.  ^b^Each min was classified as sedentary or not.  ^c^Energy expenditure was calculated according to the Actigraph formula.  ^d^Energy expenditure was calculated according to the Crouter 2-regression model. | | | |

| **Appendix Table 9 Trip-level associations between the transportation mode used and physical activity and energy expenditure (time-standardized outcomes, exclusion of trips whose start or end times were edited or reported during the survey as opposed to identified by the automatic algorithm and confirmed with the survey) (n =** **3,423 or 3,249 trips)^a^** | | | | |
| --- | --- | --- | --- | --- |
| **Transportation mode variable** | **Number of steps taken per 10min of trip**  **β (95% CI)** | **MVPA per 10min of trip (min)**  **β (95% CI)** | **Sedentary time per 10min of trip (min)^b^**  **β (95% CI)** | **Energy expenditure per 10min of trip (kcal)^c^**  **β (95% CI)** |
| **Crude classification** |  |  |  |  |
| Personal motorized vehicle | Ref. | Ref. | Ref. | Ref. |
| Public transportation | 229.0 (200.5, 257.5) | 2.0 (1.7, 2.2) | –0.8 (–1.1, –0.6) | 12.0 (10.3, 13.6) |
| Biking | 112.7 (57.2, 168.1) | 0.4 (–0.1, 0.9) | –3.1 (–3.5, –2.7) | 5.7 (2.6, 8.8) |
| Walking | 623.2 (602.4, 644.0) | 5.3 (5.1, 5.5) | –4.2 (–4.4, –4.1) | 31.0 (29.8, 32.1) |
|  |  |  |  |  |
| **Detailed classification** |  |  |  |  |
| 4-wheel motor, driving | Ref. | Ref. | Ref. | Ref. |
| 4-wheel motor, passenger | –24.9 (–72.3, 22.4) | –0.2 (–0.6, 0.2) | 0.7 (0.3, 1.0) | –0.2 (–2.9, 2.5) |
| 2-wheel motor vehicle | 1.5 (–72.0, 75.0) | –0.3 (–1.0, 0.4) | –1.8 (–2.3, –1.2) | –1.2 (–5.5, 3.1) |
| Metro | 253.8 (209.6, 298.1) | 2.3 (1.9, 2.7) | –1.1 (–1.5, –0.8) | 13.7 (11.2, 16.2) |
| Bus / coach | 174.5 (114.8, 234.3) | 1.5 (0.9, 2.0) | –1.1 (–1.6, –0.7) | 9.9 (6.6, 13.2) |
| Train | 284.8 (212.6, 357.1) | 2.2 (1.6, 2.9) | –1.0 (–1.6, –0.5) | 14.8 (10.8, 18.8) |
| Tramway | 427.3 (287.6, 567.0) | 3.8 (2.6, 5.1) | –1.8 (–2.9, –0.8) | 20.1 (12.4, 27.9) |
| Biking | 107.0 (49.9, 164.2) | 0.4 (–0.2, 0.9) | –3.1 (–3.5, –2.6) | 5.6 (2.4, 8.8) |
| Walking | 622.3 (600.2, 644.4) | 5.3 (5.1, 5.5) | –4.3 (–4.5, –4.1) | 31.0 (29.8, 32.3) |
| CI = confidence interval; MVPA = moderate to vigorous physical activity.  ^a^The multilevel linear models included a random effect at the individual level, and were not adjusted for any other covariate. The crude and the detailed transportation mode variables were introduced in separate models.  ^b^Each 5 second epoch was classified as sedentary or not (the regression coefficients were *a posteriori* converted in min of sedentary time).  ^c^Energy expenditure was calculated according to the formula of Sasaki and Freedson. | | | | |

| **Appendix Table 10 Trip-level associations between the transportation mode used and physical activity and energy expenditure (distance-standardized outcomes, exclusion of trips whose start or end times were edited or reported during the survey as opposed to identified by the automatic algorithm and confirmed with the survey) (n = 3,423 or 3,249 trips)^a^** | | | | |
| --- | --- | --- | --- | --- |
| **Transportation mode variable** | **Number of steps taken per km of trip**  **β (95% CI)** | **MVPA per km of trip (min)**  **β (95% CI)** | **Sedentary time per km of trip (min)^b^**  **β (95% CI)** | **Energy expenditure per km of trip (kcal)^c^**  **β (95% CI)** |
| **Crude classification** |  |  |  |  |
| Personal motorized vehicle | Ref. | Ref. | Ref. | Ref. |
| Public transportation | 127.8 (31.7, 223.9) | 1.0 (0.2, 1.8) | 0.1 (–1.4, 1.7) | 6.1 (0.9, 11.3) |
| Biking | 106.0 (–83.4, 295.3) | 0.6 (–1.0, 2.1) | –0.7 (–3.7, 2.3) | 3.9 (–6.4, 14.2) |
| Walking | 1106.0 (1034.5, 1177.4) | 9.0 (8.5, 9.6) | 2.4 (1.3, 3.6) | 53.1 (49.2, 56.9) |
|  |  |  |  |  |
| **Detailed classification** |  |  |  |  |
| 4-wheel motor, driving | Ref. | Ref. | Ref. | Ref. |
| 4-wheel motor, passenger | –42.9 (–207.1, 121.4) | –0.3 (–1.6, 1.0) | –0.3 (–2.9, 2.3) | –1.2 (–10.1, 7.7) |
| 2-wheel motor vehicle | –4.3 (–241.3, 232.7) | –0.1 (–2.1, 1.9) | –1.9 (–5.6, 1.9) | 0.1 (–13.1, 13.1) |
| Metro | 177.0 (22.3, 331.7) | 1.5 (0.2, 2.7) | 0.0 (–2.4, 2.5) | 8.6 (0.2, 16.9) |
| Bus / coach | 237.6 (28.0, 447.2) | 1.9 (0.2, 3.6) | 2.8 (–0.6, 6.1) | 11.6 (0.3, 22.9) |
| Train | 63.1 (–192.0, 318.1) | 0.3 (–1.8, 2.3) | –1.8 (–5.9, 2.3) | 3.8 (–10.0, 17.5) |
| Tramway | 313.2 (–182.9, 809.4) | 2.6 (–1.4, 6.6) | –1.2 (–9.2, 6.7) | 12.2 (–14.5, 38.9) |
| Biking | 99.3 (–96.9, 295.4) | 0.5 (–1.1, 2.1) | –0.8 (–3.9, 2.3) | 3.8 (–6.9, 14.4) |
| Walking | 1102.0 (1025.8, 1178.2) | 9.0 (8.4, 9.6) | 2.3 (1.1, 3.6) | 53.0 (48.9, 57.2) |
| CI = confidence interval; MVPA = moderate to vigorous physical activity.  ^a^The multilevel linear models included a random effect at the individual level, and were not adjusted for any other covariate. The crude and the detailed transportation mode variables were introduced in separate models.  ^b^Each 5 second epoch was classified as sedentary or not (the regression coefficients were *a posteriori* converted in min of sedentary time).  ^c^Energy expenditure was calculated according to the formula of Sasaki and Freedson. | | | | |

**References**

1. Thierry B, Chaix B, Kestens Y: **Detecting activity locations from raw GPS data: a novel kernel-based algorithm.** *Int J Health Geogr* 2013, **12**(1)**:**14.

2. Chaix B, Kestens Y, Perchoux C, Karusisi N, Merlo J, Meghiref K: **An interactive mapping tool to assess individual mobility patterns in neighborhood studies.** *Am J Prev Med* 2012, **43**(4)**:**440-450.

3. Sasaki JE, John D, Freedson PS: **Validation and comparison of ActiGraph activity monitors.** *J Sci Med Sport* 2011, **14**(5)**:**411-416.

4. **What is the difference among the Energy Expenditure Algorithms? https://help.theactigraph.com/entries/20744123-what-is-the-difference-among-the-energy-expenditure-algorithms. Accessed on August 23 2013.**

5. Crouter SE, Kuffel E, Haas JD, Frongillo EA, Bassett DR, Jr.: **Refined two-regression model for the ActiGraph accelerometer.** *Med Sci Sports Exerc* 2010, **42**(5)**:**1029-1037.

6. Kozey-Keadle S, Libertine A, Lyden K, Staudenmayer J, Freedson PS: **Validation of wearable monitors for assessing sedentary behavior.** *Med Sci Sports Exerc* 2011, **43**(8)**:**1561-1567.
